# Growth, instability and future outlook of livestock population and products in Bangladesh

**DOI:** 10.1371/journal.pone.0351876

**Published:** 2026-06-26

**Authors:** Abu Hayat Md. Saiful Islam, Md Mahfuzul Hasan, Mohammad Saiful Islam, Masayuki Sato

**Affiliations:** 1 Department of Agricultural Economics, Bangladesh Agricultural University (BAU), Mymensingh, Bangladesh; 2 ACASA Project, Bangladesh Agricultural Research Council, Farmgate, Dhaka, Bangladesh; 3 Graduate School of Human Development and Environment, Kobe University, Hyogo, Japan; 4 Bangladesh Livestock Research Institute, Savar, Dhaka, Bangladesh; PRISM CRO, PAKISTAN

## Abstract

In the changing agri-food system, livestock play an important role, providing not only protein and micronutrients but also organic fertilizers, employment, and women’s empowerment in many developing countries including Bangladesh. Reliable forecasting of livestock populations and products is necessary to inform policy decisions related to food security, supply, demand, and imports and exports, as well as self-sufficiency goals and associated inter- and intra-regional trade opportunities. However, robust long-term projections for livestock systems remain limited in the literature. Therefore, this study employs growth modeling and time series forecasting techniques to analyze historical trends (1961–2020) in livestock populations and products, assess their instability using the Cuddy Della instability index, and generate forecasts up to 2041 using the Box-Jenkins Autoregressive Integrated Moving Average (ARIMA) framework. Model selection was based on standard statistical criteria, including goodness of fit measures and diagnostic checks of residual behaviour. The forecasting framework assumes that historical patterns and structural relations observed in the data remain stable over the projection period and that no major structural breaks or unforeseen external shocks significantly alter underlying dynamics. The results reveal that the cubic growth model best captures long-term trends in livestock populations and products, with generally low growth rates and low instability across categories. The highest annual growth rate was observed for chicken (3.71%) among the livestock population and eggs (7.40%) among the livestock products. The analysis further indicates a persistent deficit in milk and meat production, while egg production shows comparatively stronger performance. Forecasts generated using the selected ARIMA models were ARIMA (1,2,1), ARIMA (0,2,1), and ARIMA (1,2,5), for milk, meat, and egg, respectively, suggest a continuing upward trend in production up to 2041, consistent with historical patterns and growth model estimates. Under model assumptions, milk, meat, and egg projected production levels in 2041 are estimated at yearly 4,449,394, 9,77,042, and 1,289,863 metric tons (MT), respectively, reflecting substantial increases of around 24.34%, 35.24%, and 61.51% over 2020. Although these estimates represent point forecasts and are subject to various assumptions, findings of this study will serve as invaluable evidence-based insights for livestock sector stakeholders, including researchers, policymakers, academia, livestock producers, and the feed industry.

## 1. Introduction

Bangladesh is one of the most densely populated countries in the world, with about 171.2 million people living in a landmass of 147,570 km^2^, and the predicted population is expected to grow to 196.5 million by 2040 [1,2]. The agriculture, fisheries, and livestock sectors are pillars of the economy, providing employment, income and livelihood support to a large share of the population [3]. Livestock production encompasses a diverse range of animals, including cattle, sheep, goats, poultry, and ducks. It is an essential component of Bangladesh’s complex farming system, as it provides meat, milk, eggs and supports rural livelihoods through income, employment generation and supplies draft power. The livestock sub-sector employs 20% of the entire population on a full-time basis and another 50% on a part-time basis [4,5,6]. Poultry and dairy stand out as two auspicious sectors within Bangladesh’s livestock industry, with approximately 0.3 million dairy farms and around 65–70 thousand commercial poultry farms currently in operation nationwide [7]—about 78.31% of total households rear livestock [8]. The agricultural sector’s overall contribution to Gross Domestic Product (GDP) is substantial. The livestock subsector alone accounts for 40% of the total agricultural industry, and livestock’s share of agricultural GDP is 16.52% [5]. Its contribution to the country’s GDP is about 1.9% [5]. Livestock products also have historical importance in trade and rural economies [9]. Additionally, during religious festivals such as Eid-Ul-Azha and Manosha Puja, there is a notable increase in livestock sacrifice. Moreover, the rate of animal slaughter to meet daily demand is also steadily escalating [10].

Livestock plays a vital role as a significant source of high-value animal protein for the vast majority of the global population (WHO, 2008). It contributes to rural livelihood by supporting human and social capital and enhancing natural capital accumulation [3]. A mix of microeconomic and macroeconomic variables determines the potential of livestock to drive rapid development in poverty reduction. It includes farmers’ ability to make a profit from livestock-related assets, employees’ ability to take advantage of expanded job opportunities, and consumers’ ability to benefit from more competitive pricing [11]. Meat dairy and egg products are important sources of high quality proteins, fats, and a variety of physiological and functional substances, such as micro- and trace elements and vitamins [12,13].

According to the Department of Livestock Services, per capita availability of milk, meat, and eggs in Bangladesh is 208.61 ml per day, 147.84 gm per day, and 136.01 eggs per year, respectively. In Bangladesh, about 8% of total human protein consumption comes from livestock sources [14]. Poultry meat contributes about 37% of total meat production [4]. Malnutrition remains a major policy concern, with 28% of children under five are stunted, and 10% are wasted [USAID, 2021]. Sustaining the production and supply of livestock products is therefore important for improving nutrition and food security in the country.

The livestock sector in Bangladesh has undergone structural changes over recent decades, shifting from subsistence-oriented systems towards more commercial production. This growth is driven by the rising demand for livestock products due to population growth, urbanization, and rising incomes. However, the sector faces many constraints including poor infrastructure, disease outbreaks, climate change, and limited market access [6]. Therefore, appropriate modelling of growth trends, instability and forecasting livestock population and products is essential for policy planning and sectoral development. Therefore, this study aims to estimate the current status and performance, and future outlook of the livestock sector in Bangladesh by estimating the growth rates and instability of the livestock population and products, and to forecast production of milk, meat, and eggs.

For measuring growth and forecasting, several statistical and econometric models are available and are also suitable for assessing and forecasting the performance of the agricultural and livestock sectors [15]. Mainly, two types of time series models are widely used: i) deterministic type time series models, often called growth models, such as linear, quadratic, cubic, logarithmic, exponential, compound, inverse, power, and S-shaped, and ii) stochastic time series models such as ARIMA. The former is very quick to estimate, inexpensive, and very easy to understand, and is a quite acceptable means of forecasting; the latter captures temporal dependence and stochastic variation and provides reliable forecasts and is widely used as a decision-making tool [16]. Although each approach has been used independently in previous studies, their combined application allows for a more comprehensive understanding of both structural trends and time-dependent stochastic behaviour. Therefore, this study employs both approaches to capture structural trends and time-dependent stochastic behaviour in livestock production.

Existing studies on livestock growth and forecasting remain limited, particularly in integrating deterministic and stochastic approaches within unified framework [17,18,19,20,21,22,23,24,25]. In Bangladesh, existing studies on livestock growth and forecasting are also limited. Hossain and Hassan [26] reported steady growth in livestock production with moderate variability, and Uddin et al. [27] highlighted increasing commercialization and structural transformation in the poultry and dairy sectors; however, the present study extends this evidence by identifying product-specific instability patterns and incorporating a forecasting perspective to assess long-term dynamics.. Most existing studies have not simultaneously examined growth, instability and long term forecasting using long historical datasets [28,29,30,31]. Moreover, limited attention has been given to model validation, uncertainty assessment, and the complementary use of deterministic and stochastic models in livestock forecasting applications. Particularly, SCI/Scopus index journal articles are scarce, and most of the literature is on agricultural price forecasting.

Therefore, this study contribute to this slim body of literature by using both deterministic and stochastic time series models to estimate growth, instability and forecast livestock populations and products in Bangladesh. By integrating these approaches, the study provides a more robust analytical framework for understanding historical dynamics and projecting future trends. The study provides evidence based projections of livestock production under various model assumptions. Insights from this study will empower livestock sector stakeholders, including policymakers and other value chain actors, to craft effective action plans and policies to achieve self-sufficiency in the livestock sector, thereby meeting the burgeoning demand for livestock products and ensuring food and nutrition security in Bangladesh. Furthermore, the study incorporates model validation and prediction intervals to improve the reliability and interpretability of forecasts.

The remainder of the article is organised as follows. The next section discusses data and research methods. Section 3 presents the results, and the following section 4 discusses them in the context of relevant literature. Finally, section 5 concludes with policy implications.

## 2. Methodology

### 2.1. Data

The study utilizes long-term time-series data on Bangladesh’s milk, meat, and egg production from 1961 to 2020, as well as data on the livestock populations of several main livestock species, including chicken, duck, cattle, buffalo, goats, and sheep, to achieve the study’s objectives. The data on annual figures for each of the items mentioned were obtain from the FAOSTAT [32] database of the Food and Agriculture Organization (FAO) of the United Nations (see the dataset in supporting S8 Table in S1 File). FAOSTAT is widely used in agricultural and livestock research due to its standardized data compilation procedures and broad temporal coverage, which facilitate long-run comparative and trend analyses across countries and time. Although FAOSTAT provides the most comprehensive and consistent long-term dataset available, certain limitations might be there, because of overextended time horizons, agricultural statistics may be affected by revisions in national reporting systems, changes in estimation methodologies, and improvements in data collection procedures. These factors may introduce inconsistencies in long-term series. However, FAO applies harmonization and quality control procedures to enhance comparability over time, making the dataset suitable for trend analysis and forecasting. Prior to analysis, a meticulous review and analysis of the data was conducted to identify trends, growth, and outliers in the livestock products and population scenarios obtained during 1961–2020 in Bangladesh. The variables used in this study were largely complete across the entire study period. Where minor inconsistencies or gaps were identified, standard data screening procedures were applied, including cross-verification of reported values across consecutive years to ensure temporal consistency. These preprocessing steps ensured that the final dataset maintained continuity suitable for time series modelling.

Given the long observation period, potential structural changes in the livestock sector were expected due to policy reforms, technological progress, demographic pressures, and broader socio-economic transitions. To further validate these temporal segmentations, a structural break analysis was conducted using the Bai–Perron multiple breakpoint test. The results identified several breakpoints in the livestock production series, notably around 1970, 1979, 1990, 2000, and 2011, indicating the presence of multiple structural shifts over the study period. Additionally, the Chow test confirmed the presence of significant structural change (F = 56.43, p < 0.001).

Along with, an Analysis of Variance (ANOVA) was conducted to test for differences in production levels across the defined periods. For all population and production series, the between-group and within-group degrees of freedom were 2 and 57, respectively. The ANOVA results indicate statistically significant differences in mean production across periods for all livestock population and products examined. Specifically, highly significant differences were observed for milk (F(2,57) = 170.36, p < 0.001), meat (F(2,57) = 175.49, p < 0.001), eggs (F(2,57) = 49.75, p < 0.001), chicken (F(2,57) = 171.50, p < 0.001), duck (F(2,57) = 155.84, p < 0.001), goats (F(2,57) = 171.47, p < 0.001), and sheep (F(2,57) = 77.28, p < 0.001). Cattle production also differed significantly across periods, although the magnitude of variation was comparatively smaller (F (2,57) = 6.18, p = 0.0037). The complete ANOVA results are reported in Supporting S7 Table in S1 File. The results confirmed statistically significant differences, indicating that the chosen sub-periods reflect meaningful structural changes in livestock production. These findings suggest that livestock production dynamics in Bangladesh have evolved through multiple regimes. However, direct segmentation based on every detected breakpoint would result in overly fragmented sub-periods, reducing interpretability and limiting meaningful comparisons across time. Therefore, for analytical clarity, comparability and policy relevance, the study groups these structural changes into three broader sub-periods reflecting major phases of sectoral transformation: Period I covered the years 1961–1980 considered as early development phase of the livestock sector, period II included the years 1981–2000 considered as transitional and modernization phase, and period III contained the years 2001–2020 considered as commercialization and intensification phase. These classifications align with statistically detected structural changes while maintaining interpretability and coherence in long-term analysis. Overall, the dataset preparation strategy ensures a balance between statistical rigor and practical interpretability, providing a robust foundation for subsequent growth modelling, instability analysis, and forecasting of livestock production in Bangladesh.

### 2.2. Trend and growth rate analysis

Numerous deterministic time series models, usually referred to as growth models, are used to calculate growth rate. Linear, logarithmic, quadratic, cubic, exponential, compound, inverse, and power are among the most popular growth models. Some of those models are time-dependent, while others are not. We must evaluate each model’s goodness-of-fit or selection criterion in order to choose the best growth model [33,34]). Five main models—linear, logarithmic, quadratic, cubic, and compound are used in this study. These models’ mathematical formulations are provided below (Table 1).

**Table 1:** Mathematical forms of different growth models.

Name of the model	Functional form	Meaning
Linear	Y = α + βt + ε	α, β, γ, δ are the coefficients of the model and t represent time
Logarithmic	Y = α + β ln(t)+ε	
Quadratic	Y = α + βt + γ$t^{\text{2}}$+ε	
Cubic	Y = α + βt + γ$t^{\text{2}}$+ δ$t^{\text{3}}$+ε	
Compound	Y = α$\beta^{t}$ε	

Source: Hasan et al. [35].

### 2.3. Model selection criteria

Model selection was based on multiple complementary criteria rather than reliance on a single statistical measure. These included goodness-of-fit indicators (R² and RMSE), statistical significance of estimated parameters, visual inspection of fitted versus observed trends, and parsimony considerations. This multi-criteria approach was adopted to reduce the risk of selecting over-parameterized models, particularly in long-run time series contexts where flexible functional forms may artificially improve in-sample fit. According to the model selection criteria, the model is a better fit if it has higher values of adjusted R^2^. On the other hand, the model is more fit if its values for RMSE, AIC, BIC are lower [34,36,33]. Detailed model comparison results are presented in Supporting S1 Table in S1 File. And best fitted statistics are shown in Table 2.

**Table 2:** Best fitted statistics.

Variable	Selected Model	Adj R²	RMSE	AIC	BIC
Chicken	Cubic	0.983	10,389	1290.10	1300.57
Duck	Cubic	0.994	1,087,488	1848.20	1858.67
Cattle	Cubic	0.281	1,584,701	1893.38	1903.85
Buffalo	Cubic	0.926	91,615	1551.31	1561.79
Goat	Cubic	0.989	1,864,964	1912.92	1923.39
Sheep	Cubic	0.909	150,894	1611.19	1621.66
Milk	Cubic	0.969	170,474	1625.83	1636.30
Meat	Cubic	0.981	22,798	1384.41	1394.88
Egg	Cubic	0.953	38,339	1446.78	1457.25

### 2.4 Estimation of growth rates

After testing the diagnostic test or the goodness-of-fit, the researcher found that the cubic model is the best for calculating the growth rate for the selected items. The cubic specification was selected not as a theoretically imposed biological law, but as an empirical approximation capable of capturing potential nonlinearities, including turning points, accelerations, and decelerations commonly observed in long-term agricultural time series including livestock growth over time. This flexibility is particularly relevant in the context of structural transformations in agricultural systems driven by technological change, policy interventions, market expansion, and demographic pressures. But, in this research both cubic and compound growth model were used to identify complementary insights into livestock production dynamics. While the cubic model generally provides a better statistical fit, the compound model offers a more interpretable measure of average growth. Cubic function is presented in equation 1


$$\begin{array}{c} \text{Y }=\;\alpha \;+\;\beta \text{t }+\;{\gamma t}^{\text{2}}+\;\delta t^{\text{3}}\;+\;\epsilon \end{array}$$ (1)

After differencing, we got


$$\begin{array}{c} ((\beta +\text{2}\gamma \text{t}+\text{3}\delta \text{t}\text{2})/\text{y})\;*\text{100} \end{array}$$ (2)

For greater accuracy, researchers also consider Compound Annual Growth Rates (CAGRs), as they help compare models and yield appropriate results. A suitable functional form specified by [37] was used to estimate the compound growth rate using the compound growth model [37]. Unlike the cubic specification, the compound model assumes a constant proportional growth rate over time and is widely used in agricultural and economic analyses for summary growth estimation [18; 21]. Based on the [38], the CAGR function is estimated by fitting a semi-log trend equation as follows:


$$\begin{array}{c} {\;{\text{Y}}_{\text{t}}\;=\;\alpha \;\beta }^{t}\;\epsilon \text{t} \end{array}$$ (3)

The Compound growth rate was obtained by transforming the above function to logarithmic form as follows:


$$\begin{array}{c} \text{lnY }=\text{ ln}\alpha +\text{ t}(\text{ln}\beta ) \end{array}$$ (4)

Where Y is the dependent variable, t is the period in years, and α and β are the coefficients. To determine the CAGR, we divide the ending value by the beginning value. After that, the result should be multiplied by one and divided by the number of years. The outcome is then reduced by 1 and multiplied by 100, as shown in the following equation.


$$\begin{array}{c} \text{CAGR }=\;((\text{BV}/\text{EV})\;\text{1}/\text{n }-\text{1})\;\times \text{100} \end{array}$$ (5)

Furthermore, autocorrelation is a common feature in time series data. To examine the presence of serial correlation in the growth models, the Durbin–Watson (DW) statistic was employed. The results for key livestock products (milk, meat, and eggs) are presented in Table 3. The initial DW values (0.09, 0.077, and 0.075, respectively) are substantially below the benchmark value of 2, indicating strong positive autocorrelation in the residuals. To address this issue, the Prais–Winsten transformation was applied. This method, based on generalized least squares (GLS), corrects for first-order autoregressive [AR(1)] errors and provides more efficient parameter estimates. After applying the transformation, the DW statistics increased to 1.37, 1.88, and 2.06 for milk, meat, and eggs, respectively, suggesting that autocorrelation has been substantially reduced. Further diagnostic analysis across all livestock components (S2 Table in S1 File) also indicates the presence of positive autocorrelation in the initial models, which is consistent with the nature of long-term time series data. The Durbin–Watson statistic and autocorrelation checks, were conducted before and after applying the Prais–Winsten correction which ensure that serial correlation was adequately addressed. It is important to distinguish the role of the Prais–Winsten estimator from that of the ARIMA modelling framework used in this study. The Prais–Winsten procedure is applied within the deterministic growth models solely as an econometric correction technique for serial correlation in regression residuals. In contrast, ARIMA models are used independently as a stochastic time series approach that explicitly models temporal dependence through autoregressive and moving average components.

**Table 3:** Durbin-Watson test value for livestock products.

Production	R^2^	Adj. R^2^	Durbin-Watson test value	
			Before transformation	After transformation
Milk	0.971	0.969	0.09	1.37
Meat	0.982	0.981	0.077	1.88
Egg	0.955	0.953	0.075	2.06

Source: Authors’ calculation.

### 2.5. Instability analysis

A quick analytical method for identifying fluctuations or instability in a time series of data is the instability index [39]. If data are dispersed around a negative or positive trend line, this approach adjusts the coefficient of variation. The Coefficient of Variation (CV) and Cuddy and Della Valle’s instability indices (CDVI) were employed as measures of variability to calculate an instability index [40]. The formula suggested by Cuddy-Della Valle was used to measure instability, which is given as follows:


$$\begin{array}{c} \text{CDVI }=\text{ CV }\sqrt{(\text{1}-\;{\text{adj}.\text{R}}^{\text{2}}\;)\;} \end{array}$$ (6)

Where CV is the coefficient of variation (CV = (Standard Deviation/Mean)*100) [18]. Adj R^2^ = Coefficient of determination from a time trend regression (linear) adjusted by the number of degrees of freedom [41]. The range of CDVI 0–15 denotes low instability, 15–30 denotes medium instability, and greater than 30 means high instability.

### 2.6. Forecasting Analysis: ARIMA model

A variety of statistical methods and time-series forecasting models are available including ARIMA, Exponential Smoothing State Space models (ETS), Theta model and the Prophet model. ARIMA is well-suited for non-stationary univariate time series and is particularly effective in capturing autocorrelation structures after differencing, making it appropriate for long annual datasets without strong seasonal patterns. ETS models are primarily designed to capture level, trend, and seasonal components through weighted smoothing and generally perform well in short- to medium-term forecasting with relatively stable patterns. The Prophet model, in contrast, is designed to handle complex seasonality, holiday effects, and nonlinear trend shifts, and is typically more effective in high-frequency or highly dynamic data environments ([42]; [43,44,45]. To choose the best-fit model for forecasting milk, meat, and egg production, the study evaluated the Auto Regressive Integrated Moving Average (ARIMA) via the Box-Jenkins methodology and deterministic models such as the cubic. Given that the present study uses long-term annual data (1961–2020) with no pronounced seasonal variation and a dominant trend component, ARIMA was selected as the primary forecasting framework due to its theoretical suitability, interpretability, and strong performance in modelling non-stationary economic and agricultural time series. Nevertheless, the alternative approaches are acknowledged as useful extensions for future comparative forecasting research. In practice, the ARIMA model requires extensive time-series data spanning at least 50 years [46]. Extensive time-series observations ensure the accuracy of the model’s forecasting. The ARIMA model has a wide range of applications in the agriculture sector, including wheat [47], jute [46], rice [48], and potatoes [49]. The stochastic model (ARIMA) is best for forecasting the future values of a time series. It can capture linear associations between previous and current data points, as well as errors or residuals in the data [23]. A stochastic process is declared as stationary when the mean and covariance are constant. For a non-seasonal time series, the model is denoted ARIMA (p, d, q). It has three main components, which are given below:

I. Autoregressive process (AR): Autoregressive process is the first component of the ARIMA model, where AR (p) refers to the autoregressive process of order p. It establishes a connection between past and present values of the observations. The relation can be written as:

$$\begin{array}{c} x_{t}=\;\mu +\varepsilon_{t}+\;\theta_{\text{1}}\varepsilon_{t-\text{1}}+\;\theta_{\text{2}}\varepsilon_{t-\text{2}}\pm ---+\;\theta_{q}\varepsilon_{t-q}--- \end{array}$$ (7)

Where, X_t_ is the observed value of time t, µ is the mean of the time series, ɛ_t_ is the white noise error term at time t, and $\emptyset_{\text{1}}$, $\emptyset_{\text{2}}$, …, $\emptyset_{q}$ are the parameters to be estimated.

II. Degree of difference (I): In the ARIMA model, the degree of difference I (d) means the order of difference (d). It means the number of times the raw observations are differentiated. By differentiation, non-stationary data is transformed into stationary data.
III. Moving average (MA): The final component of the ARIMA model is the moving average process MR (q) of order q, which establishes the relationship between the current and past errors or residuals [50; 23]. It can be expressed as:

$$\begin{array}{c} X_{t}=\;\emptyset_{\text{1}}X_{t-\text{1}}+\;\emptyset_{\text{2}}X_{t-\text{2}}\pm ---+\;\emptyset_{p}X_{t-p}+\varepsilon_{t}---- \end{array}$$ (8)

Where, X_t_ is the observed value of time t, Ø_1_, Ø_2,_…, Ø_p_ are the autoregressive parameters, and ɛ_t_ is the white noise error term at time t.

Thus, the ARIMA model is a combination of AR (p), I (Degree of Differentiation), and MA (q) processes. Finally, the ARIMA (p, d, q) model can be written as:


$$\begin{array}{c} X_{t}=\;\mu +\varepsilon_{t}+\;\theta_{\text{1}}\varepsilon_{t-\text{1}}+\;\theta_{\text{2}}\varepsilon_{t-\text{2}}+---+\;\theta_{q}\varepsilon_{t-q}+\emptyset_{\text{1}}X_{t-\text{1}}+\;\emptyset_{\text{2}}X_{t-\text{2}}+---+\;\emptyset_{p}X_{t-p}+\varepsilon_{t}----\; \end{array}$$ (9)

The p, d, and q values were determined using the Autocorrelation Function (ACF), which is tentatively accepted as the p and q parameters for an ARIMA (p, d, q) process. For a non-stationary series, the data are differenced to make the series stationary. The theoretical PACF has nonzero partial autocorrelations at lags 1,2...n and has zero partial autocorrelation at all lags. To use the model, data must be transformed from non-stationary to stationary using the differencing method [51]. The Dickey-Fuller unit root test tests the following hypothesis: the time series of livestock products is non-stationary. If the computed Tau (ͳ) is greater than or equal to the DF critical value, then the null hypothesis is rejected; otherwise, it is accepted [52]. On the other hand, we also conducted a Cubic model to predict the future production of milk, meat, and eggs. It helped to compare the predicted values from the ARIMA and the Cubic model. To assess the predictive performance of the selected ARIMA models, out-of-sample validation was conducted using a train–test split approach. Approximately 80% of the observations were used to estimate the models (training set), while the remaining 20% were reserved for validation (testing set) (S4 Table in S1 File). The models estimated from the training data were used to generate forecasts for the testing period, and forecast accuracy was evaluated using standard measures, including Root Mean Square Error (RMSE), Mean Absolute Error (MAE), and Mean Absolute Percentage Error (MAPE). In addition, residual diagnostics were assessed using the Ljung–Box test to ensure that the models adequately captured the underlying data structure. The normality of residuals was assessed using the Jarque–Bera test (S3 Table in S1 File). The results indicate deviations from normality; however, this does not affect model validity, as ARIMA models primarily require the absence of autocorrelation, which is confirmed by the Ljung–Box test. Furthermore, to formally compare the predictive accuracy of competing forecasting models, the Diebold–Mariano test was employed. This test evaluates whether the difference in forecast errors between two models is statistically significant. In this study, the predictive performance of the selected ARIMA models was compared against a benchmark naïve model using out-of-sample forecasts obtained from the test dataset. The null hypothesis of the Diebold–Mariano test is that the expected loss differential is zero, implying equal predictive accuracy between the two models. A statistically significant test statistic indicates that one model provides superior forecasting performance over the other. In this study, the test was conducted using squared error loss, and the results were used to assess whether the selected ARIMA models significantly outperform the benchmark model in terms of forecast accuracy. Diebold–Mariano test results for forecast accuracy comparison are shown in S5 Table in S1 File.

## 3. Results

Descriptive statistics for livestock population and production variables over the period 1961–2020 are presented in Table 4. The results reveal substantial variation across variables, reflecting differences in scale, growth patterns, and structural dynamics within the livestock sector. The mean values indicate that duck and goat populations are the largest among livestock categories, with averages exceeding 27 million, followed by cattle at approximately 23.5 million. In contrast, smaller-scale variables such as chicken, buffalo, and sheep exhibit comparatively lower mean values. Among production variables, milk production shows a relatively high mean (2.08 million), while meat and egg production remain lower in magnitude. The standard deviation values highlight considerable variability across most variables, particularly for duck, goat, and milk, indicating expansion and increasing fluctuations over time. The coefficient of dispersion is especially pronounced for egg production, suggesting higher instability relative to other variables. All variables exhibit positive skewness, indicating right-skewed distributions characterized by upward trends and occasional sharp increases. This is particularly evident for cattle (skewness = 2.05) and egg production (skewness = 1.78), suggesting stronger growth surges and asymmetry in these series. Kurtosis values further indicate differences in distributional shape. Cattle (kurtosis = 9.39) and egg production (kurtosis = 5.55) display leptokurtic behavior, implying the presence of extreme values and higher volatility. In contrast, other variables exhibit relatively moderate kurtosis, suggesting more stable distributions. The observed non-normality and heterogeneity support the use of flexible time-series approaches capable of accommodating structural changes and dynamic evolution in the data. In particular, such properties justify the application of deterministic growth models to capture long-run trends and Autoregressive Integrated Moving Average (ARIMA) models to account for temporal dependence and stochastic variation. Furthermore, the presence of dispersion and non-normality across variables underscores the importance of employing differencing and diagnostic testing in subsequent time-series analysis to ensure stationarity and model adequacy.

**Table 4:** Descriptive statistics of livestock population and products (1961–2020).

Variable	Mean	Standard Deviation (SD)	Minimum	Maximum	Skewness	Kurtosis
Chicken	122,046	82,805	16,000	296,602	0.74	2.17
Duck	27,051,550	14,608,343	7,000,000	59,716,000	0.59	2.19
Cattle	23,496,454	1,934,035	19,850,000	31,741,008	2.05	9.39
Buffalo	871,553	349,104	429,000	1,493,000	0.57	2.04
Goat	27,283,336	18,158,391	7,600,000	60,026,848	0.48	1.72
Sheep	1,136,152	516,382	490,000	2,274,314	0.72	2.38
Milk	2,077,825	1,009,431	915,460	3,791,748	0.46	1.68
Meat	385,739	173,207	168,900	722,405	0.57	1.88
Egg	178,497	182,182	21,000	798,592	1.78	5.55

**Source:** Authors’ calculation based on FAOSTAT data.

Overall, these statistical properties indicate non-constant mean and variance over time, supporting the presence of non-stationarity in the series. This justifies the application of differencing and time series models, such as ARIMA, in subsequent analysis.

### 3.1. Trend and growth in livestock population

**Fig 1:**
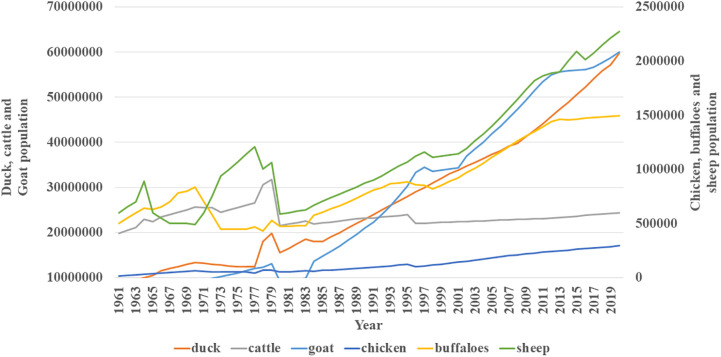
Trend of livestock population.

**Fig 2:**
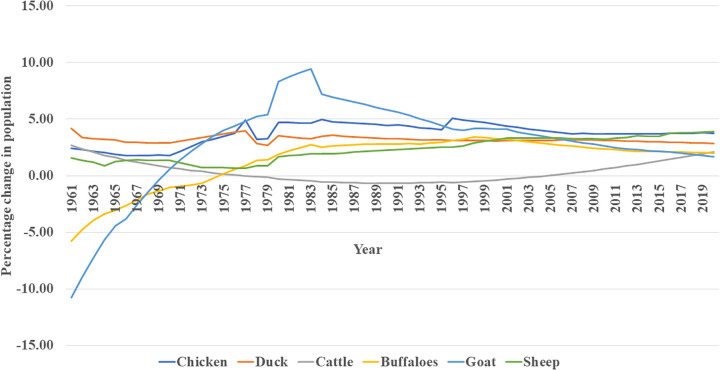
Cubic growth rate of the livestock population.

This section analysed the cubic growth rate, compound growth rate, and instability for chickens, ducks, cattle, buffaloes, goats, and sheep across three sub-periods and the complete period. The cubic and compound growth rates for the populations of chickens, ducks, cattle, buffalo, goats, and sheep throughout three observational periods have shown various ups and downs, as shown in Table 5. The population growth rates of sheep, goats, buffalo, and chicks have also accelerated in the cubic models during the second and third sub-periods. Except for sheep, it fell during the third sub-period. In the first two sub-periods, the duck’s growth rate remained at 3.27, but it decreased in the last period. After dropping to −0.56% in the second sub-period from 0.86%, the cubic growth rate for cattle increased to 0.74% in the third sub-period. In addition, compound growth for chicken, duck, and cattle declined in the second period from 6.08, 4.08, and 0.41% to 4.48, 3.53, and 0.10%, respectively, and decreased further in the third period, except for cattle. Additionally, in the compound growth model, the growth rates of sheep and buffaloes are negative in the first sub-period; however, they may improve in the following sub-periods. When examining the compound and cubic growth rates over the entire time period, the chicken had the highest values, at 3.71% and 15.72%, respectively. Duck and goat came in at the second and third ranks, respectively, in terms of growth rates. Cattle experience the slowest growth rates, 0.35% and 1.04%, respectively (see Figs 1 and 2).

**Table 5:** Cubic and compound growth rate for livestock population and products.

Period	Model	Population						Products		
		Chicken	Duck	Cattle	Buffalo	Goat	Sheep	Milk	Meat	Egg
1961−80		2.70	3.27	0.86	−1.30	−0.14	1.11	0.13	0.68	10.77
1981−00	Cubic	4.61	3.27	−0.56	2.85	5.93	2.32	3.93	3.12	3.54
2001−20		3.83	3.05	0.74	2.45	2.71	3.47	1.95	2.51	7.89
Overall		3.71	3.20	0.35	1.33	2.83	2.30	2.00	2.10	7.40
1961−80		6.08	4.08	0.41	−0.26	0.96	−0.10	1.20	1.07	5.58
1981−00	Compound	4.48	3.53	0.10	3.18	6.64	3.22	3.65	3.76	5.09
2001−20		3.73	2.88	0.43	2.45	2.82	3.50	1.92	2.29	7.38
Overall		4.76	3.50	0.31	1.79	3.49	2.21	2.26	2.37	6.02

Source: Authors’ calculation.

The cubic growth of the livestock population is depicted in Fig 2, and it is evident that the growth rates of the cattle, buffalo, and goats vary more than those of the other three animals. During the 60-year, the growth rates of chicken, duck, and sheep were almost comparable and never went negative. In contrast, those of cattle, buffalo, and goats fluctuated between negative and positive growth rates. At the beginning of the year, the goats’ growth rate was negative, but it has since increased steadily. Following that, it grew faster, reaching a higher rank in 1983 at 9.43%, before also declining more slowly. Over the course of the year, buffalo followed the same trend line. There is an upward trend line for all animals’ production throughout the year. Duck, goat, buffalo, and sheep are pretty similar in nature. They are moving upward with large fluctuations, but cattle show a gradual increase, like a chicken. Among those things, chicken production came last.

### 3.2. Trend and growth in production of milk, meat and eggs

Cubic growth rate, compound growth rate, and instability in milk, meat, and egg production were evaluated for three sub-periods and the entire period. Over three observational periods, the cubic and compound growth rates for milk, meat, and egg production are all positive. However, some ups and downs are evident in Table 5. For both models, the growth rate of meat and milk production has accelerated during the second sub-period (1981–2000). However, it fell in the third sub-period (2001–2020). For Egg, the cubic growth rate improved to 7.89% in the third sub-period after falling to 3.54% in the second sub-period from 10.77%. Similarly, compound growth for eggs has also fallen in the second sub-period, 1981–2000 (5.09%), but increased in the third sub-period. While looking at the growth rates of the overall time period, eggs have the highest cubic and compound growth rates, such as 7.40% and 19.95%, respectively, followed by meat and milk. In contrast, milk has the lowest growth rates at 2.00% and 7.05%, respectively. The egg growth rate shows greater fluctuations than the other two items. Milk and meat growth rates were nearly identical during the 60-year. They overlap, but the egg production growth rate appears to differ: it was 50.98% in 1961, then declined to 0.18% in the 1980s, and then increased at a minimal rate.

**Fig 3:**
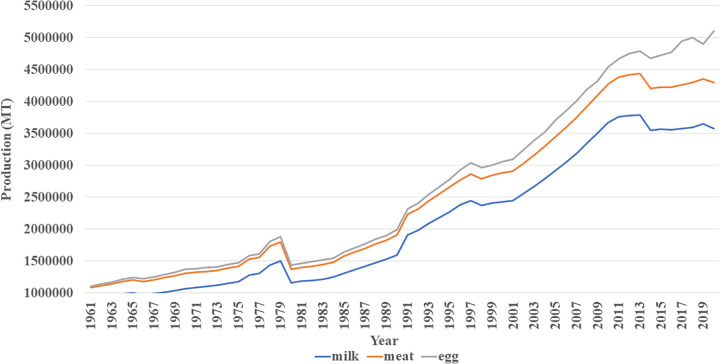
Trend of livestock products: milk, meat, and eggs.

**Fig 4:**
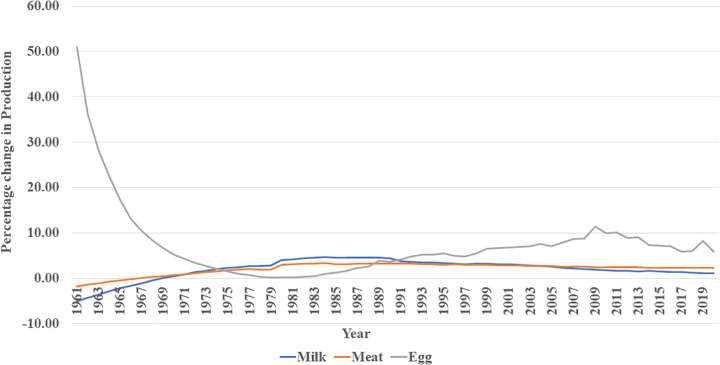
Cubic growth rate of milk, meat, and eggs.

Over the course of the year, there is an upward trend line, and egg production remained higher than milk and meat production (see Figs 3 and 4). Among those, the milk production trend and growth position are third. The percentage changes in output during the previous 60 years are shown in Table 6. Egg production increases by 3703%, followed by milk and meat with 291 and 328%, respectively.

**Table 6:** Percentage changes in the production of milk, meat, and eggs.

Production	1961	2020	% Change
Milk	915460	3578373	291%
Meat	168900	722405	328%
Eggs	21000	798592	3703%

Source: Authors’ calculation.

### 3.3. Instability in livestock population and products

The Cuddy-Della Valle instability index is also calculated for chicken, duck, cattle, buffalo, goat, and sheep. The population of chickens, ducks, cattle, buffaloes, and sheep has shown a lower CDVI in period III than in period II, and even in period I. It has diminished throughout the third sub-period. However, goat instability is higher in period II than in period I. Given the instability of the overall time period, the CDVI values for chicken, duck, cattle, buffalo, goat, and sheep range from 0 to 15. Similarly, the Cuddy-Della Valle instability index is applied to milk, meat, and egg production, as well. The second sub-period has seen increased instability in the production of milk, meat, and eggs. Among the three sub-periods, instability has diminished throughout the third sub-period, except for eggs. Given the instability of the overall time period, the CDVI values for milk and meat are in the low instability range, and egg has an overall CDVI of 22.12%, which falls in the medium instability category. The mean, standard deviations (SD), and coefficient of variation are also shown (Table 7).

**Table 7:** Instability for the livestock population and products.

Study period	Stat.	Population						Products		
**Chicken**	**Duck**	**Cattle**	**Buffalo**	**Goat**	**Sheep**	**Milk**	**Meat**	**Egg**
1961-1980	SD	15304	2878097	2954796	130299	1572330	244401	163798	33107	15444
	Mean	47684	12528050	24596451	582400	9713550	766650	1108630	227345	51520
	CV	32.09	22.97	12.01	22.37	16.19	31.88	14.77	14.56	29.98
	CDVI	4.18	1.78	10.19	6.09	1.70	9.62	2.60	2.01	6.50
1981-2000	SD	24585	5333980	666008	145993	8848666	193318	487944	73888	42959
	Mean	91959	23925550	22670311	732610	22448051	890850	1798844	326184	104028
	CV	26.73	22.29	2.94	19.93	39.42	21.70	27.13	22.65	41.30
	CDVI	3.49	1.73	2.49	5.42	4.13	6.55	4.78	3.12	8.95
2001-2020	SD	46855	8106790	608088	197267	8071916	354937	434222	80374	188294
	Mean	226496	44701050	23222600	1299650	49688407	1750956	3325999	603688	379944
	CV	20.69	18.14	2.62	15.18	16.25	20.27	13.06	13.31	49.56
	CDVI	2.70	1.40	2.22	4.13	1.70	6.12	2.30	1.84	10.74
Overall	SD	82805	14608343	1934035	349104	18158391	516382	1009430	173207	182182
	Mean	122047	27051550	23496454	871553	27283336	1136152	2077824	385739	178497
	CV	67.85	54.00	8.23	40.06	66.55	45.45	48.58	44.90	102.06
	CDVI	8.85	4.18	6.98	10.90	6.98	13.71	8.55	6.19	22.13

Source: Authors’ calculation.

### 3.4. Forecasting of livestock products in Bangladesh

**Fig 5:**
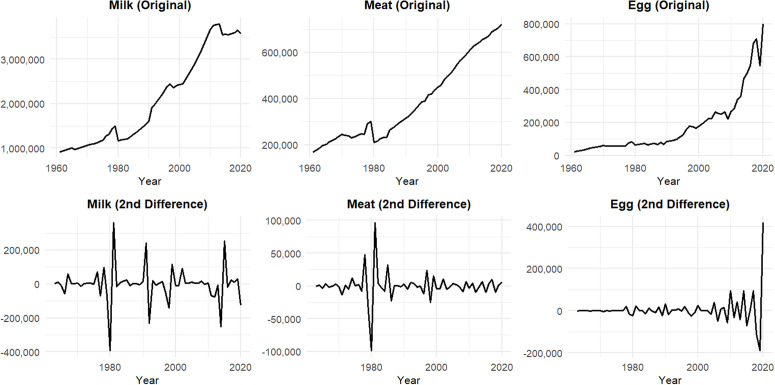
Original and after differencing time series plot for Milk, Meat and Egg.

The original and differenced time-series data for milk, meat, and egg production from 1961 to 2020 were displayed through line graphs. The purpose of this visual comparison is to assess the stationarity of each series, which is required for ARIMA modelling. The original series exhibits strong upward trends, confirming non-stationarity, while the second-differenced series show stable fluctuations around the mean, indicating that differencing successfully achieved stationarity (Fig 5).

The Augmented Dickey-Fuller (ADF) test results are used to formally evaluate whether the milk, meat, and egg production series are stationary. The ADF statistics and corresponding p-values for the original series indicate non-stationarity. After second differencing, the ADF values become significant for milk and meat, confirming stationarity, whereas egg remains near-stationary. These results justify the use of an ARIMA model with a differencing order 𝑑 = 2 (Table 8).

**Table 8:** Augmented Dickey-Fuller Test.

Product	ADF Test	Dickey-Fuller	Lag order	p-value
Milk	Without differencing	−2.42	3	0.40
	2^nd^ differencing	−4.94		0.01
Meat	Without differencing	−1.28		0.86
	2^nd^ differencing	−5.71		0.01
Egg	Without differencing	0.24		0.99
	2^nd^ differencing	−3.24		0.08

Source: Authors’ calculation.

**Fig 6:**
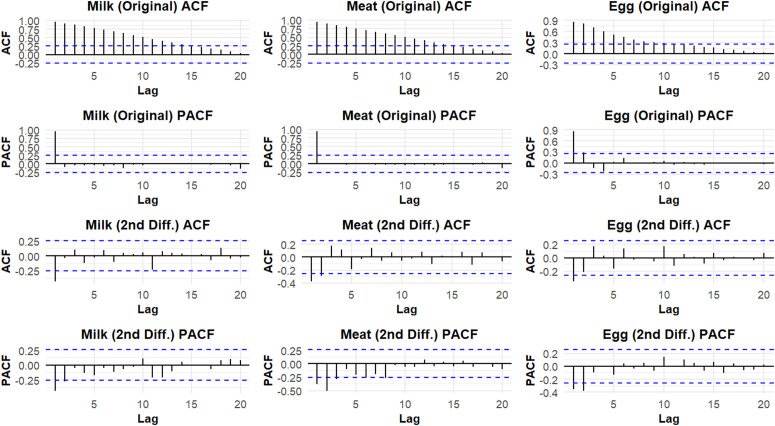
ACF and PACF curves of the original and differenced series.

Moreover, the autocorrelation function (ACF) and partial autocorrelation function (PACF) plots for the original and differenced series of milk, meat, and egg production were visualized using ACP and PACF plots. These diagnostic plots guide the identification of appropriate AR (p) and MA (q) orders for ARIMA models. The original series shows slow decay in the ACF, typical of non-stationary trends. In contrast, the differenced series exhibits clearer cut-off or limited-lag significance patterns, which help determine suitable ARIMA parameter combinations (Fig 6).

**Fig 7:**
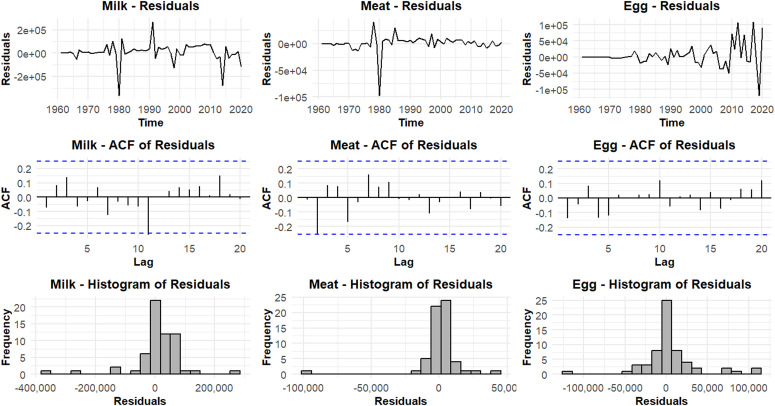
Residuals of Milk, Meat and Egg.

A well-fitted model should produce residuals that behave like white noise, with no visible autocorrelation or systematic patterns. The residual plots confirm that residuals are randomly distributed around zero, supporting the adequacy of the selected ARIMA specifications for forecasting (Fig 7).

The model fit statistics for several ARIMA model configurations tested for milk, meat, and egg production are summarized in Table 9. The comparison relies on key diagnostic indicators, including R², RMSE, MAE, MAPE, and BIC. By evaluating these criteria collectively, the study identifies ARIMA (1, 2, 1) for milk, ARIMA (0, 2, 1) for meat, and ARIMA (1, 2, 5) for eggs as the best-performing models (Table 9).

**Table 9:** Model fit statistics.

ARIMA Model	Milk			Meat			Egg			
	1.2.1	1.2.0	0.2.0	0.2.0	0.2.1	1.2.1	1.2.5	1.2.1	1.2.2	1.2.3
Stationary R²	0.11	−0.1	−0.37	−0.88	0.01	0.01	0.47	0.23	0.26	0.25
R²	0.99	0.99	0.99	0.98	0.99	0.99	0.96	0.95	0.95	0.95
RMSE	83239	92173	102691	21318	15522	15521	34463	41567	40676	41096
MAPE	2.58	2.87	3.03	3.59	2.54	2.54	10.09	9.46	9.28	8.95
MAE	48966	51655	53413	10249	7491	7492	19994	20784	20395	20139
MaxAPE	31.51	36.56	33.88	47.24	46.77	46.76	36.58	40.81	41.11	40.27
MaxAE	366274	424937	393796	98794	97815	97791	119960	222520	224144	219562
Normalized BIC	24.94	25.02	25.15	22.11	21.6	21.67	23.57	23.58	23.61	23.69
Ljung-Box Q Statistics	13.1	17.15	22.45	25.76	12.22	12.24	7.09	10.07	8.77	9.01
DF	18	18	18	18	18	18	18	18	18	18
Sig.	0.79	0.51	0.21	0.11	0.84	0.83	0.99	0.93	0.96	0.96
Number of Outliers	3	1	2	2	1	1	2	1	1	1

Source: Authors’ calculation.

**Fig 8:**
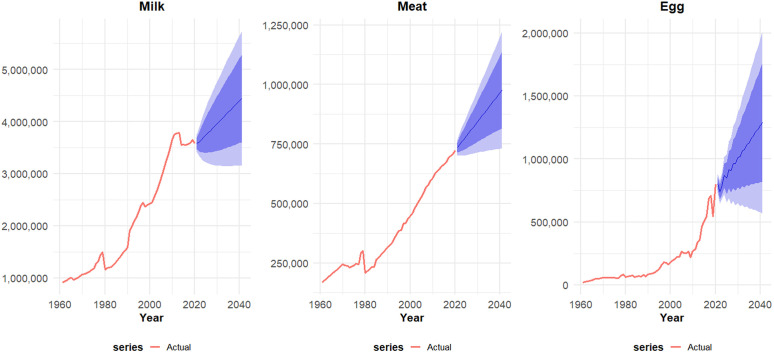
Forecasting of Milk, Meat and Egg.

The 21-year forecasts (2021–2041) for milk, meat, and egg production were generated using the best-fitted ARIMA models. The results of the ARIMA model forecasting are presented in Fig 8 and Table 10. The graphs include historical observations, fitted values, and projected trends. The results indicate a consistent upward trajectory for all three products, with egg production showing the steepest projected growth. The forecasted patterns reflect the continuation of long-term structural growth in livestock production (Fig 8).

**Table 10:** Forecasting result (Milk–Meat–Egg by Cubic and ARIMA).

Year	Milk		Meat		Egg	
	Cubic	ARIMA	Cubic	ARIMA	Cubic	ARIMA
2021	3881933	3585840	762199	734531	756056	803525
2022	3914907	3618331	778623	746656	806988	739146
2023	3943905	3658714	795112	758782	860395	787840
2024	3968755	3701587	811660	770907	916337	867915
2025	3989284	3745244	828257	783033	974872	847845
2026	4005321	3789150	844896	795158	1036060	914523
2027	4016694	3833134	861567	807284	1099959	906057
2028	4023231	3877142	878264	819409	1166627	962684
2029	4024759	3921158	894976	831535	1236125	962925
2030	4021107	3965177	911696	843661	1308511	1012009
2031	4012102	4009197	928417	855786	1383843	1018784
2032	3997573	4053216	945128	867912	1462181	1062209
2033	3977347	4097236	961822	880037	1543584	1073886
2034	3951253	4141256	978491	892163	1628110	1113064
2035	3919119	4185275	995127	904288	1715818	1128420
2036	3880771	4229295	1011720	916414	1806767	1164411
2037	3836039	4273315	1028263	928540	1901016	1182527
2038	3784751	4317335	1044748	940665	1998625	1216128
2039	3726733	4361354	1061165	952791	2099651	1236315
2040	3661815	4405374	1077508	964916	2204154	1268121
2041	3589824	4449394	1093766	977042	2312192	1289863

Source: Authors’ calculation.

The numerical forecasts of milk, meat, and egg production for 2021–2041, generated using both the Cubic and ARIMA models, are described in Table 10. Presenting both models result allows comparison between deterministic and stochastic forecasting approaches. ARIMA forecasts show a more pronounced increase, especially for eggs, while the cubic model produces relatively smoother projections. The dual presentation helps validate the robustness of the predicted trends. According to ARIMA, if current growth rates continue, milk, meat, and egg output in Bangladesh will reach 4449394, 977042, and 4646447 in 2041, respectively, whereas using cubic forecast production would yield 3589824, 1093766, and 2312192, respectively, in 2041 (Table 10). Forecasted value with 95% confidence interval estimated through ARIMA model for Milk, Meat and Egg are shown in S6 Table in S1 File.

## 4. Discussion

The study assessed the growth performance, instability, and future production trends of major livestock products in Bangladesh, including milk, meat, and eggs, alongside the population dynamics of key livestock species. The findings reveal substantial variation in growth patterns across products and species, with apparent shifts in production trajectories over the three observed sub-periods. The growth analysis showed that production of all three livestock products exhibited favourable cubic and compound growth rates throughout the study period. However, the rates and directions of change varied across sub-periods. Milk and meat production experienced accelerated growth during the second sub-period (1981–2000), while egg production grew most rapidly during the first and third sub-periods. Similar to crop-based studies, in which productivity improvements stemmed from hybrid varieties and increased input use, the rise in livestock product output in Bangladesh may also be associated with improvements in breed performance, expanded commercial farming, greater feed availability, and growing private-sector participation in the poultry and dairy value chains.

Despite positive long-term trends, growth in milk and meat production slowed during the most recent sub-period (2001–2020), reflecting structural challenges such as feed scarcity, rising production costs, disease burdens, and market volatility. Conversely, egg production recovered during the final sub-period, driven by rapid expansion in commercial poultry enterprises and improved management practices. As seen in other livestock-producing countries, the poultry sector has responded more efficiently to rising demand due to its shorter production cycle, higher feed conversion efficiency, and greater technological adaptability. Samad [53] notes that rising population and income levels have significantly boosted demand for milk and meat, thereby enhancing production capacity.

To evaluate whether Bangladesh will be able to meet future national demand for milk, meat, and eggs, we have estimated projected demand and supply for 2041. Table 11 showed, 2020–21 fiscal year demand and production comparison with projected demand and production in 2041. The demand for each product was calculated based on per-capita recommended intake, predicted population size, and standard consumption conversion factors. For eggs, an average egg weight of 57 grams (0.057 kg) was used. Similar methods were applied to estimate milk and meat demand. The table highlights the existing demand and supply differences and how these gaps may change by 2041, even under projected increases in production. This comparison is essential for identifying potential deficits or surpluses and for guiding policy interventions to achieve self-sufficiency in livestock-derived food products.

**Table 11:** Current (2020−21) and projected demand (2041) and production.

Products (MT)	Current (2020−21)			Projected (2041)		
	Demand	Production	Difference	Demand	Production	Difference
Milk	2071433	3578373	1506939	2451923	4449394	1997471
Meat	2429834	722405	−1707429	2876156	977042	−1899114
Egg	771472	798592	27119	913179	1289863	376684

**Note:** The weight of an egg can vary, but the average weight for a large egg with a shell is approximately 57 grams (0.057 kg). We multiply the current consumption amount (per day) *population (predicted population) *per egg weight (ton)*365. Just like an egg, we can also calculate Milk and Meat demand (MT).

Forecasts of future dynamics in milk, meat, and egg production in Bangladesh using both cubic trend and ARIMA models have some merits. The use of these two distinct approaches enables a meaningful comparison between deterministic long-term trend extrapolation and stochastic time-series forecasting, thereby improving the robustness and interpretability of the results. Similar dual-model approaches have been recommended in agricultural forecasting literature to account for both structural trends and short-run fluctuations [52,54]. Both models consistently project increasing milk, meat, and egg production over the forecast horizon (2021–2041), indicating sustained expansion of the livestock and poultry sectors. This upward trend aligns with previous studies that attribute growth in Bangladesh’s livestock production to rising population, urbanization, income growth, and changing dietary preferences toward animal-source foods [55,56]. However, the magnitude of projected growth varies substantially between the two models—the ARIMA model forecasts markedly higher production levels than the cubic model, particularly for egg production. By 2041, ARIMA projections suggest milk, meat, and egg outputs of 4449394, 977042, and 1289863, respectively, compared with cubic estimates of 3,589,824, 1,093,766, and 2,312,192. The stronger growth indicated by the ARIMA model reflects its ability to capture recent acceleration, cyclical behaviour, and stochastic variations embedded in the historical data. Previous empirical studies have shown that ARIMA models often outperform deterministic trend models in short- to medium-term agricultural forecasting when growth momentum is strong [57,58,59].

The divergence between the two models is especially pronounced for egg production, suggesting that the poultry sector has experienced a rapid recent expansion that heavily influences ARIMA forecasts. This finding is consistent with evidence that Bangladesh’s poultry industry has grown faster than other livestock subsectors due to lower capital requirements, shorter production cycles, and strong market demand [4,60,61]. However, reliance on ARIMA alone may overestimate long-term output if current growth rates are not sustained due to constraints such as feed shortages, disease outbreaks, environmental pressures, or policy changes. In contrast, the cubic model provides smoother, more conservative projections that reflect long-term structural trends rather than short-term volatility. Such models are useful for long-range planning where gradual technological progress and resource constraints are expected to moderate growth [62]. The cubic forecasts may therefore represent a lower-bound or baseline scenario, particularly relevant for policymakers concerned with sustainability and risk management.

The combined use of cubic and ARIMA models offers a plausible range of future production outcomes rather than a single forecast. This approach enhances confidence in the predicted upward trends while acknowledging uncertainty regarding growth intensity. For policy formulation, the ARIMA projections may be interpreted as an optimistic scenario under continued investment and productivity gains, whereas the cubic forecasts provide a more cautious outlook. Effective planning for food security, infrastructure development, and environmental sustainability in Bangladesh’s livestock sector should account for both scenarios to build resilience against future uncertainties.

Furthermore, the livestock population dynamics also exhibited uneven growth patterns. Chicken, duck, and sheep populations grew steadily with low instability, while cattle, buffalo, and goat populations showed more pronounced fluctuations, including negative growth in some years. These fluctuations reflect the sensitivity of ruminant livestock to feed shortages, disease outbreaks, and climate-related stressors. The sharp instability in goat and buffalo populations during earlier sub-periods mirrors findings from other regions where small ruminants are highly vulnerable to environmental stress and management constraints. Forecasting results illustrate important policy implications. Both ARIMA and cubic models suggest continued growth in livestock product production, with substantial surpluses emerging in milk and eggs by 2041. However, the meat sector shows a persistent and growing deficit, similar to the yield gaps reported in staple crop studies. Without strategic intervention, Bangladesh will continue to rely heavily on meat imports to meet domestic demand. The sustained deficit in meat production indicates structural bottlenecks involving inadequate fodder supply, limited genetic improvement, poor veterinary coverage, and low adoption of improved management systems. In the South Asian context, these bottlenecks are compounded by the persistent burden of transboundary animal diseases. Foot and mouth disease (FMD), for instance, remains endemic across the region and continues to constrain cattle and meat productivity; its widespread distribution and recurring emergence in Pakistan illustrate the scale of disease pressure facing ruminant livestock systems regionally [63]. As observed in other livestock-dependent countries, productivity-enhancing technologies such as high-yielding breeds, improved pasture management, disease control, and farmer training are essential to narrow this gap. Crossbreeding and improved cattle management practices have resulted in higher yields, with crossbred cows producing an average of 1,838 liters of milk over 266 days, compared with local breeds [64].

Instability patterns further support the need for targeted policy actions. Milk and meat production displayed moderate long-term instability, while eggs exhibited medium instability due to the poultry industry’s vulnerability to disease outbreaks and market shocks. This vulnerability is well-documented in the South Asian context: viral diseases such as Infectious Bursal Disease (Gumboro), which has caused recurrent and severe losses in commercial poultry flocks across the region, represent a concrete mechanism through which production stability is disrupted and contribute to output volatility in the region [65]. Egg production has been more responsive to market changes, with significant growth during the first and third sub-periods, likely due to lower production costs and higher consumer acceptance, according to Huque and Khan [66]. In contrast, livestock population instability was mostly low, suggesting that population growth is relatively stable, but productivity improvements remain crucial. The findings align with global evidence that stability in animal numbers does not necessarily translate into proportional gains in product output unless feed, health, and management interventions improve.

The large projected surpluses in milk and eggs indicate opportunities for value addition, processing, and export expansion. Similar to the rapid growth in vegetable production driven by consumer demand and market incentives in other settings, Bangladesh’s poultry and dairy sectors could benefit from improved cold-chain infrastructure, investment in processing plants, and stronger market linkages. This is particularly pressing given that rapid commercialization of the dairy sector brings heightened food safety risks: studies from the South Asian region have documented significant prevalence of antimicrobial-resistant pathogens such as Listeria monocytogenes in raw milk and dairy products, underscoring the need for robust pathogen control, improved processing standards, and cold-chain integrity as preconditions for safe market expansion [67]. For meat, however, the study highlights the urgency of strengthening production systems through improved genetics, disease-resistant breeds, feed innovation, and effective extension services. Encouraging expansion of the livestock area, enhancing sustainable fodder production, and developing climate-resilient management practices are vital to raising productivity. Finally, the study’s findings show that, despite overall positive growth, sustained improvement in the livestock sector will require continued investment in veterinary services, improved breed development, enhanced feed resources, and private-sector-driven innovation. However, government initiatives aimed at strengthening the dairy sector have facilitated growth, including investments in feed production and extension services [68]. The forecast results emphasize that, without major structural reforms, deficits in meat production will persist even as the milk and egg sectors expand rapidly. This underscores the need for a differentiated policy strategy: productivity intensification for dairy and poultry, and systemic reinforcement for the meat subsector.

**Fig 9:**
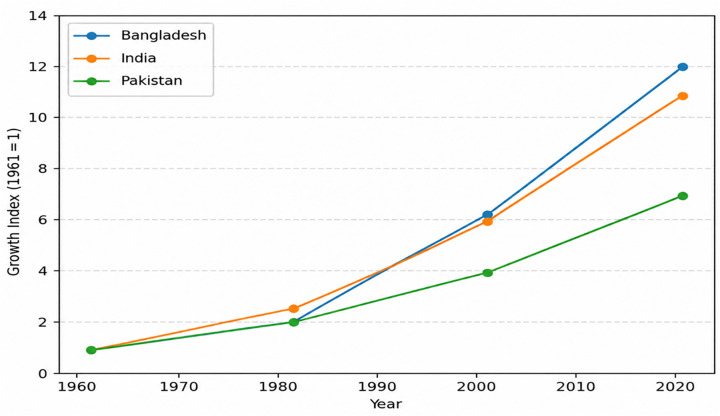
Comparative livestock growth trends in South Asia (1961–2020). Note: Values are indexed (1961 = 1) to illustrate relative growth patterns across countries based on long-term trends from FAOSTAT.

The results are also broadly consistent with previous empirical studies on livestock and agricultural growth in South Asia, which also report sustained expansion driven by rising demand for animal-source foods, population growth, and gradual commercialization, as documented in regional statistics (Fig 9). However, the structural pathways and stability of this growth differ markedly across countries. India’s livestock sector shows relatively more stable and institutionally supported expansion, largely underpinned by cooperative dairy systems and productivity-enhancing technological change [69,70,71,72,73]. Pakistan, by contrast, experiences more pronounced output volatility due to persistent constraints such as feed shortages, climatic variability, and disease risks [74,75,76,77,78]. Bangladesh reflects an intermediate but more dynamic pattern, characterized by rapid commercialization—particularly in poultry and dairy—alongside higher sensitivity to structural breaks, disease shocks, and input market fluctuations [79,80,81] [82,83,84]. [85]

Overall, while livestock growth across South Asia shares common demand-driven foundations, differences in institutional development and production systems critically shape variability, resilience, and long-term predictability, underscoring the need for country-specific policy responses rather than generalized regional strategies. From a forecasting perspective, these differences imply that model reliability and predictive stability vary significantly across South Asia and suggest that regionally uniform forecasting models may be inappropriate; instead, adaptive and country-specific approaches that account for structural heterogeneity and instability are essential for improving long-term predictive accuracy and policy relevance.

## 5. Conclusions and Policy Implications

Many significant findings emerged from analysing trends, growth patterns, and variations in the production of milk, meat, and eggs, as well as the population dynamics of key livestock species in this study. In the beginning, it is evident that over the three assessed periods, milk, meat, and egg production have demonstrated positive growth rates in both the Cubic (2.00, 2.10, and 7.40%, respectively) and Compound (2.26, 2.37, and 6.02%, respectively) growth models. Nonetheless, fluctuations in growth rates underscore the importance of closely examining market dynamics, particularly in egg production. The rising trend in egg production relative to milk and meat suggests a possible shift in customer preferences toward poultry products. Growing public knowledge of healthful eating practices may influence this change, potentially affecting red meat consumption. Therefore, further research is needed to assess the potential impact of dietary changes on the consumption of various livestock products. Furthermore, the examination of rates of increase in livestock populations reveals differences among species. Cattle grow more slowly than chickens do, suggesting that the livestock industry has different patterns. Chickens grow the fastest. The production of meat and milk is expected to rise, suggesting both industries have the potential to grow and attract investors. However, the ARIMA model’s significant prediction of a rise in egg production points to potential opportunities for expansion in poultry farming, indicating a changing face of animal production. In conclusion, all parties in the agricultural sector must have a clear understanding of the growth patterns, trends, and fluctuations in livestock output. The findings have several important implications for livestock sector development. First, the persistent growth trend suggests that continued investment in livestock production systems can play a significant role in ensuring food security and rural livelihoods. However, the observed instability highlights the need for policies that enhance resilience, particularly in response to disease outbreaks, input price volatility, and climatic stress. Second, the presence of structural changes implies that policy interventions should be adaptive rather than static, with regular reassessment of sectoral strategies based on emerging trends. Third, while forecasts indicate positive long-term growth, policymakers should treat these projections as scenario-based guidance rather than deterministic outcomes. Finally, strengthening data systems, veterinary services, and market infrastructure will be essential to reduce volatility and support sustained growth in the livestock sector. Policymakers and farmers can overcome hurdles and seize new opportunities to ensure sustainable growth and development in the cattle sector by regularly monitoring market dynamics and consumer preferences.

Despite significant efforts to produce robust results, some caveats are acknowledged. First, both deterministic growth models and ARIMA models rely on simplifying assumptions regarding trend stability and temporal dependence. Second, long-term forecasts are subject to increasing uncertainty, particularly over extended horizons such as 2041, due to potential structural shifts not captured in historical data. However, this limitation is inherent in all univariate time-series models, especially in systems influenced by policy shifts, environmental shocks, and technological change. Third, although FAOSTAT provides the most comprehensive available dataset, long-term agricultural data may still be affected by revisions in reporting practices and measurement inconsistencies over time. Additionally, external factors such as policy interventions, disease outbreaks, climate variability, and market disruptions may introduce structural changes that could alter future trajectories. Despite these constraints, the study makes an important contribution by integrating deterministic trend analysis, instability measurement, structural break detection, and stochastic forecasting within a unified framework. This combined approach provides a more comprehensive understanding of livestock sector dynamics than methods relying on a single modelling strategy. It also highlights both the growth potential and the inherent uncertainty associated with long-term projections in agricultural systems. This paper advocates further research to analyse the factors that could influence the demand and supply dynamics of livestock products, including the burgeoning population, evolving consumer preferences, climatic and non-climatic shocks and advancements in livestock production technologies.

## Supporting information

S1 File S1 Table. Comparative Performance of Alternative Growth Models for Livestock Components**. S2 Table. Autocorrelation Diagnostics for Livestock Models. S3 Table. Jarque–Bera test for normality of ARIMA residuals. S4 Table. Performance of selected ARIMA models for livestock production. S5 Table. Diebold–Mariano test results for forecast accuracy comparison. S6 Table. Forecasted value with 95% confidence interval. S7 Table. ANOVA results for livestock production variables across defined structural periods. S8. Livestock population and product data set.**
(ZIP)
