# Supplementary material for: Growth, instability and future outlook of livestock population and products in Bangladesh
Source: PLoS One. 2026 Jun 26;21(6):e0351876. doi: 10.1371/journal.pone.0351876 (PMC13308833; doi:10.1371/journal.pone.0351876)
Supplement: S1 File — Comparative Performance of Alternative Growth Models for Livestock Components. S2 Table. Autocorrelation Diagnostics for Livestock Models. S3 Table. Jarque–Bera test for normality of ARIMA residuals. S4 Table. Performance of selected ARIMA models for livestock production. S5 Table. Diebold–Mariano test results for forecast accuracy comparison. S6 Table. Forecasted value with 95% confidence interval. S7 Table. ANOVA results for livestock production variables across defined structural periods. S8. Livestock population and product data set. (ZIP) [file pone.0351876.s001.zip › S3 Table.docx]

**S3 Table. Jarque–Bera test for normality of ARIMA residuals**

| **Variable** | **JB Statistic** | **p-value** | **Interpretation** |
| --- | --- | --- | --- |
| Milk | 206.72 | 0.000 | Residuals not normally distributed |
| Meat | 1854.08 | 0.000 | Residuals not normally distributed |
| Egg | 45.52 | 0.000 | Residuals not normally distributed |
