# Supplementary material for: Growth, instability and future outlook of livestock population and products in Bangladesh
Source: PLoS One. 2026 Jun 26;21(6):e0351876. doi: 10.1371/journal.pone.0351876 (PMC13308833; doi:10.1371/journal.pone.0351876)
Supplement: Attachment — Submitted filename: reviewer comments.docx [file pone.0351876.s002.docx]

**Growth, Instability and Forecasting of Livestock Population and Products in Bangladesh:**

**Analysis Using Best Fitting Deterministic and Stochastic Models**

**Reviewer Comments**

**Title**

The title is excessively long and contains methodological detail (“Best Fitting Deterministic and Stochastic Models”) that reads more like methods than a research focus.

The phrase “Best Fitting” is interpretative and should be avoided unless rigorously justified against alternative frameworks.

The title could be simplified to improve clarity and readability without loss of scientific meaning.

**Abstract**

The abstract reports many numerical results but lacks clear emphasis on methodological limitations and assumptions underlying long-term forecasting (to 2041).

The statement that outputs are “an invaluable resource” is overstated and not supported by validation or uncertainty assessment.

Forecast values are presented without confidence intervals or prediction uncertainty, which is critical for forecasting studies.

The abstract implies robustness but does not mention model validation strategy.

**Introduction**

The introduction is overly descriptive and contains extensive background statistics that do not directly build the research gap.

The literature gap is weakly justified; claims of scarcity of studies are not sufficiently supported by systematic referencing.

Some statements are outdated or inconsistently referenced, and several citations appear disconnected from the forecasting objective.

The rationale for combining deterministic growth models with ARIMA is stated but not theoretically justified.

**Methodology**

**Data**

Data source is limited to FAOSTAT without discussion of potential data quality issues or structural breaks over a 60-year period.

No explanation is provided for handling missing values, revisions, or consistency across historical data series.

Splitting the time series into three sub-periods appears arbitrary; no statistical justification is provided.

**Growth Models**

Model selection is based heavily on goodness-of-fit statistics (R², RMSE, etc.), which may favor overfitting (especially cubic models) in long-term trend data.

The theoretical justification for preferring cubic growth models over alternatives is insufficient.

Growth rate derivation from the cubic function is presented mathematically but lacks interpretation of economic or biological meaning.

**Autocorrelation Treatment**

The use of Prais–Winsten transformation for growth models is reported, but the rationale for applying it alongside ARIMA (which already accounts for autocorrelation) is unclear.

Durbin–Watson statistics reported before transformation are extremely low, suggesting severe autocorrelation that may indicate model misspecification rather than simply a correction issue.

**Forecasting / ARIMA**

Model identification relies largely on visual ACF/PACF interpretation; a clearer systematic model selection procedure is needed.

Forecast validation (e.g., train–test split, out-of-sample testing) is not described.

Forecast horizon to 2041 (21 years) is long relative to model structure and lacks uncertainty discussion.

Egg series stationarity remains borderline after differencing (p = 0.08), yet ARIMA modeling proceeds without sufficient justification.

**Results**

**Descriptive Statistics**

Some descriptive statistics appear inconsistent (e.g., mean/SD scales and formatting), suggesting possible data reporting issues.

Interpretation of skewness and kurtosis is superficial and does not contribute meaningfully to later modeling.

**Growth and Instability Analysis**

Results are largely descriptive without statistical testing of differences between periods.

The interpretation of instability categories (low/medium/high) lacks discussion of practical implications.

Figures are difficult to interpret due to scaling and presentation style.

**Forecasting Results**

Model comparison relies heavily on in-sample fit metrics; no predictive performance evaluation is shown.

Residual diagnostics are described qualitatively but lack formal statistical tests (e.g., Ljung–Box).

Forecast plots and uncertainty bands are missing, limiting interpretability of future projections.

**Discussion**

Discussion mainly restates results rather than critically interpreting findings.

Limited comparison with existing forecasting studies beyond descriptive references.

No discussion of structural changes (policy shifts, disease outbreaks, climate factors) that could invalidate long-term forecasts.

Policy implications are presented as definitive despite methodological uncertainty.

**Conclusions**

Conclusions are stronger than warranted by the methods and validation presented.

Forecast outputs are framed as actionable policy guidance without acknowledging model limitations.

Key limitations (model assumptions, uncertainty, data constraints) are insufficiently emphasized.

**Figures and Tables**

Several figures are visually cluttered and not publication-ready (small labels, unclear axes).

Some plots appear to be screenshots rather than high-resolution figures.

Table formatting and decimal precision are inconsistent across results.

**References**

Reference list appears inconsistent in formatting and citation style.

Several citations seem outdated or not directly relevant to forecasting methodology.

Some methodological references are very recent but insufficiently integrated into methodological reasoning.

Citation consistency (author names, years, punctuation) should be carefully revised.
