# Supplementary material for: Growth, instability and future outlook of livestock population and products in Bangladesh
Source: PLoS One. 2026 Jun 26;21(6):e0351876. doi: 10.1371/journal.pone.0351876 (PMC13308833; doi:10.1371/journal.pone.0351876)
Supplement: Attachment — Submitted filename: Response to Reviewers.docx [file pone.0351876.s004.docx]

PONE-D-26-07519

**Growth, Instability and Forecasting of Livestock Population and Products in Bangladesh:**

**Analysis Using Best Fitting Deterministic and Stochastic Models**

**Reviewer Comments**

**Title**

The title is excessively long and contains methodological detail (“Best Fitting Deterministic and Stochastic Models”) that reads more like methods than a research focus.

The phrase “Best Fitting” is interpretative and should be avoided unless rigorously justified against alternative frameworks.

The title could be simplified to improve clarity and readability without loss of scientific meaning.

**Response:** Thank you for your thoughtful and constructive comment regarding the title. We agree that the original title was overly long and included methodological detail that may reduce clarity and readability. We also acknowledge that the phrase “Best Fitting” could be interpreted as subjective without explicit comparative justification. In response, we have revised the title to make it more concise and focused on the core research theme while removing interpretative language and excessive methodological emphasis.

**Revised title:**
“Growth, Instability and Future Outlook of Livestock Population and Products in Bangladesh”

We believe this revised version improves clarity, avoids methodological over-specification, and aligns more closely with standard academic conventions.

**Abstract**

The abstract reports many numerical results but lacks clear emphasis on methodological limitations and assumptions underlying long-term forecasting (to 2041).

The statement that outputs are “an invaluable resource” is overstated and not supported by validation or uncertainty assessment.

Forecast values are presented without confidence intervals or prediction uncertainty, which is critical for forecasting studies.

The abstract implies robustness but does not mention model validation strategy.

**Response:** We thank the reviewer for these valuable and constructive comments. In response, we have substantially revised the abstract to improve clarity and scientific rigor. Specifically, we have (i) reduced overstated language regarding the usefulness of the results, (ii) explicitly acknowledged that the forecasts are conditional point estimates, (iii) clarified the assumptions underlying the long-term forecasting framework, including the stability of historical relationships over time, (iv) removed implicit claims of robustness by emphasizing the limitations of ARIMA-based long-term forecasting, and (v) briefly indicated the use of standard model selection and diagnostic validation procedures. These revisions have been made to ensure that the abstract more accurately reflects the methodological constraints and uncertainty associated with long-term projections, in line with the reviewer’s suggestions.

**Introduction**

The introduction is overly descriptive and contains extensive background statistics that do not directly build the research gap.

The literature gap is weakly justified; claims of scarcity of studies are not sufficiently supported by systematic referencing.

Some statements are outdated or inconsistently referenced, and several citations appear disconnected from the forecasting objective.

The rationale for combining deterministic growth models with ARIMA is stated but not theoretically justified.

**Response:** We sincerely thank the reviewer for the thorough and constructive evaluation of the Introduction section. We have carefully revised the manuscript to address all concerns raised, with substantial improvements in clarity, focus, and methodological justification. The specific changes made are outlined below:

We have significantly streamlined the Introduction by removing several detailed background statistics and contextual descriptions that were not directly contributing to the identification of the research gap or the study objectives. These sections have been condensed to maintain relevance and improve narrative flow, while retaining only essential information necessary to establish the importance of the livestock sector in Bangladesh. The justification of the research gap has been substantially revised. Previously, general and unsupported statements regarding the scarcity of literature have been replaced with a more precise and evidence-based discussion. We now explicitly highlight that existing studies rarely integrate growth analysis, instability measurement, and long-term forecasting within a unified framework, particularly for Bangladesh. We have reviewed and refined the cited literature to ensure stronger alignment with the forecasting focus of the study. Outdated or less directly relevant references have been removed or rephrased, and the discussion now better reflects recent contributions in livestock growth and forecasting research. We have strengthened the justification for combining deterministic growth models with stochastic ARIMA models. The revised text clearly explains that deterministic models are used to capture long-term structural trends, while ARIMA models are applied to model short-term stochastic variations and improve forecasting performance. This provides a clearer conceptual foundation for the hybrid modelling approach adopted in the study. Sections that were overly descriptive or not directly linked to the research objectives—particularly those discussing detailed consumption breakdowns and subsector comparisons—have been removed to improve focus and conciseness. The final part of the introduction has been rewritten to better align with the study aims, clearly stating the objectives related to growth estimation, instability analysis, and forecasting of livestock production in Bangladesh. We believe these revisions have substantially improved the clarity, focus, and academic rigor of the introduction section, making it more suitable for publication and more aligned with the reviewer’s recommendations.

**Methodology**

**Data**

Data source is limited to FAOSTAT without discussion of potential data quality issues or structural breaks over a 60-year period.

No explanation is provided for handling missing values, revisions, or consistency across historical data series.

Splitting the time series into three sub-periods appears arbitrary; no statistical justification is provided.

**Response:** Thank you for this important and insightful comment. We appreciate the opportunity to clarify and strengthen the data and methodological aspects of the manuscript. First, regarding the use of FAOSTAT data, we have now added a discussion in the revised manuscript acknowledging potential limitations, including issues related to data quality, revisions, and consistency over long historical periods. We also note that FAOSTAT is widely used in longitudinal agricultural studies due to its standardized data collection and harmonization procedures, making it suitable for cross-period analysis despite inherent limitations.

Second, we have clarified how missing values and data consistency were handled. The dataset used in this study is largely complete for the variables analyzed; where minor inconsistencies or gaps occurred, we applied standard data cleaning procedures and cross-checked series to ensure temporal consistency. These steps are now explicitly described in the revised methodology section. Third, concerning the division of the time series, we agree that arbitrary segmentation should be avoided. To address this, we conducted a formal structural break analysis using the Bai–Perron multiple breakpoint test. The test results indicate the presence of multiple structural changes over the study period. However, dividing the data strictly according to all detected breakpoints would result in numerous short segments, reducing interpretability and limiting meaningful comparison across periods. Therefore, for analytical clarity and to retain comparability, we grouped the identified structural changes into three broader and policy-relevant sub-periods (1961–1980, 1981–2000, and 2001–2020). These intervals reflect major phases of structural transformation in Bangladesh’s livestock sector while remaining consistent with the underlying statistical evidence. This justification has now been clearly incorporated into the revised manuscript. We believe these revisions improve the transparency, rigor, and credibility of the study. Thank you again for your valuable feedback.

**Growth Models**

Model selection is based heavily on goodness-of-fit statistics (R², RMSE, etc.), which may favor overfitting (especially cubic models) in long-term trend data.

The theoretical justification for preferring cubic growth models over alternatives is insufficient.

Growth rate derivation from the cubic function is presented mathematically but lacks interpretation of economic or biological meaning.

**Response:** Thank you for this insightful comment. We appreciate the concern regarding model selection, potential overfitting, and the need for stronger theoretical grounding and interpretation. First, we agree that relying solely on goodness-of-fit measures such as R² and RMSE may favor more flexible functional forms (e.g., cubic models), particularly in long-term time series data. In the revised manuscript, we have clarified that model selection was not based exclusively on these criteria. We now explicitly discuss the risk of overfitting and complement goodness-of-fit statistics with visual inspection of trends, parsimony considerations, and consistency of estimated growth patterns over time. Second, regarding theoretical justification, we would like to clarify that the cubic and compound growth models serve different but complementary purposes rather than acting as strict competitors. The compound growth model provides a standard and easily interpretable measure of average growth over time, which is widely used in agricultural and economic studies. In contrast, the cubic model is employed as a flexible functional form to capture potential non-linearities, turning points, and structural changes in livestock population dynamics that cannot be adequately represented by simpler models. We have revised the manuscript to better articulate this rationale and to emphasize that the cubic specification is used as an empirical approximation of complex growth trajectories rather than a theoretically driven biological model. Third, we acknowledge that the interpretation of growth rates derived from the cubic function was previously insufficient. In the revised version, we have expanded the discussion to provide clearer economic and sectoral interpretation of the estimated trends, including phases of acceleration, deceleration, and possible stagnation in livestock growth. These are linked to broader developments in Bangladesh’s livestock sector, such as technological changes, policy shifts, and market expansion, thereby enhancing the practical relevance of the results. Overall, these revisions improve the transparency of model selection, address concerns about overfitting, and strengthen the linkage between empirical results and their economic interpretation. We thank the reviewer for helping us improve this aspect of the manuscript.

**Autocorrelation Treatment**

The use of Prais–Winsten transformation for growth models is reported, but the rationale for applying it alongside ARIMA (which already accounts for autocorrelation) is unclear.

Durbin–Watson statistics reported before transformation is extremely low, suggesting severe autocorrelation that may indicate model misspecification rather than simply a correction issue.

**Response:** Thank you for this important and insightful comment. We appreciate the opportunity to clarify the rationale behind our methodological choices and to strengthen this aspect of the manuscript. First, we would like to emphasize that the Prais–Winsten transformation and ARIMA models were employed for **distinct but complementary purposes**, rather than redundantly addressing the same issue. The Prais–Winsten estimator was applied within the deterministic growth models to correct for first-order serial correlation in the regression residuals. This correction is necessary to obtain efficient parameter estimates and valid statistical inference when estimating trend-based growth equations. In contrast, ARIMA models were used as part of a **stochastic time series framework** specifically designed for modeling temporal dependence structures and generating forecasts. Unlike the deterministic models, ARIMA does not impose a predefined functional form on growth but instead captures the underlying data-generating process through autoregressive and moving average components. We have revised the manuscript to make this distinction more explicit.

Second, regarding the very low Durbin–Watson statistic observed prior to transformation, we agree that this indicates severe autocorrelation. In the revised manuscript, we now clarify that this finding not only justifies the use of Prais–Winsten correction but also signals potential limitations of simple deterministic specifications when applied to long-term time series data. To address this concern, we complemented the analysis with additional diagnostic checks, including residual autocorrelation functions and model adequacy tests, to ensure that the corrected models are statistically appropriate. Finally, we have expanded the discussion to acknowledge that strong autocorrelation may reflect deeper dynamic structures in the data, which further supports the use of ARIMA models as a complementary approach for capturing such dynamics and improving forecasting performance. These revisions improve the clarity of the methodological framework and reinforce the robustness of our empirical strategy. We thank the reviewer for highlighting this important issue.

**Forecasting / ARIMA**

Model identification relies largely on visual ACF/PACF interpretation; a clearer systematic model selection procedure is needed.

Forecast validation (e.g., train–test split, out-of-sample testing) is not described.

Forecast horizon to 2041 (21 years) is long relative to model structure and lacks uncertainty discussion.

Egg series stationarity remains borderline after differencing (p = 0.08), yet ARIMA modeling proceeds without sufficient justification.

**Response:** Thank you for this valuable and detailed comment. We have carefully revised the manuscript to improve the transparency, rigor, and justification of the ARIMA modeling and forecasting approach. First, regarding model identification, we agree that relying solely on visual inspection of ACF and PACF plots may be insufficient. In the revised manuscript, we clarify that these plots were used only as an initial diagnostic tool. Final model selection followed a more systematic and comparative approach, where multiple candidate ARIMA specifications were estimated and evaluated using a range of information criteria and forecast accuracy measures, including BIC, RMSE, MAE, and MAPE. We also note that the auto.arima() procedure was used as a preliminary guide, but the final model choice was based on overall diagnostic performance, parsimony, and residual adequacy.

Second, we acknowledge the importance of forecast validation. In response, we have incorporated an explicit validation strategy in the revised manuscript by conducting out-of-sample forecasting using a train–test split of the data. Model performance was evaluated based on predictive accuracy over the hold-out sample, thereby strengthening the credibility of the forecasting results. Third, concerning the long forecast horizon (up to 2041), we agree that uncertainty increases substantially over time. We have now explicitly acknowledged this limitation in the manuscript and clarified that the projections should be interpreted as indicative long-term trends rather than precise forecasts. In addition, we have incorporated and reported forecast intervals to better reflect the increasing uncertainty associated with longer horizons. Finally, regarding the stationarity of the egg production series, we recognize the reviewer’s concern. The series exhibited strong non-stationarity at levels and only borderline stationarity after first differencing (p = 0.08). To ensure robustness, we applied second-order differencing (d = 2), which resulted in a stable and well-behaved series suitable for ARIMA modeling. The selected ARIMA(1,2,5) model was supported by diagnostic checks, including residual independence and stability, as well as superior performance across multiple evaluation criteria. We have clarified this justification more explicitly in the revised manuscript. Overall, these revisions enhance the methodological rigor, transparency, and reliability of the forecasting analysis. We thank the reviewer for helping us strengthen this important component of the study.

**Results**

**Descriptive Statistics**

Some descriptive statistics appear inconsistent (e.g., mean/SD scales and formatting), suggesting possible data reporting issues.

Interpretation of skewness and kurtosis is superficial and does not contribute meaningfully to later modeling.

**Response:** Thank you for this helpful observation. We have carefully reviewed and revised the descriptive statistics table to ensure consistency in scale, formatting, and overall presentation. All summary measures (mean, standard deviation, minimum, and maximum) have been rechecked for accuracy and are now reported in a uniform and coherent manner. In addition, we acknowledge that the earlier discussion of skewness and kurtosis was limited and not sufficiently linked to the subsequent analysis. In the revised manuscript, we have refined this section by either streamlining the interpretation or explicitly connecting these distributional characteristics to the modeling strategy—particularly in relation to data transformation, non-linearity, and potential deviations from normality assumptions in time-series modeling. These revisions improve both the clarity and analytical relevance of the descriptive statistics section. We thank the reviewer for highlighting this issue.

**Growth and Instability Analysis**

Results are largely descriptive without statistical testing of differences between periods.

**Response:** Thank you for this important comment. We agree that relying solely on descriptive comparisons across sub-periods is not sufficient without formal statistical validation. To address this concern, we have strengthened the methodological rigor in two ways. First, we applied the Bai–Perron multiple breakpoint test to formally identify structural changes in the time series and to ensure that the periodization is empirically grounded rather than arbitrary. Second, to statistically validate differences across the identified sub-periods, we conducted one-way ANOVA tests to examine whether mean differences in key variables across periods are statistically significant. The results confirm that the observed variations across sub-periods are statistically meaningful, thereby supporting the period-wise interpretation of growth and instability patterns. Together, these approaches strengthen the empirical foundation of the period classification and enhance the robustness of the growth and instability analysis.

**Discussion**

Discussion mainly restates results rather than critically interpreting findings.

Limited comparison with existing forecasting studies beyond descriptive references.

No discussion of structural changes (policy shifts, disease outbreaks, climate factors) that could invalidate long-term forecasts.

Policy implications are presented as definitive despite methodological uncertainty.

**Response:** Thank you for this insightful and constructive comment. We agree that the discussion section required strengthening in terms of critical interpretation, engagement with the broader literature, and explicit consideration of structural and contextual factors affecting long-term forecasts. In response, we have substantially revised the discussion section to move beyond restating results and to provide a more critical interpretation of the findings. Specifically, we now more explicitly situate our results within the existing forecasting literature on livestock and agricultural time series, highlighting both consistencies and divergences with previous studies, rather than only providing descriptive comparisons. We have also expanded the discussion to incorporate important structural and contextual drivers that may influence long-term forecasting performance. These include major policy shifts in the livestock sector, disease outbreaks affecting production systems, and climate variability, all of which can introduce structural breaks and uncertainty into long-run projections. This addition helps to more clearly acknowledge the limitations of deterministic forecasting over extended horizons.

Finally, we have revised the policy implications section in conclusion to reflect methodological uncertainty more carefully. Rather than presenting implications as definitive outcomes, we now frame them as conditional and scenario-based suggestions, emphasizing that long-term forecasts should be interpreted as indicative trends rather than precise predictions. This adjustment improves the balance between analytical findings and their practical interpretation. We thank the reviewer for these valuable suggestions, which have significantly improved the depth and rigor of the Discussion section.

**Conclusions**

Conclusions are stronger than warranted by the methods and validation presented.

Forecast outputs are framed as actionable policy guidance without acknowledging model limitations.

Key limitations (model assumptions, uncertainty, data constraints) are insufficiently emphasized.

**Response:** Thank you for this important and constructive comment. We agree that the original Conclusions section required a more cautious interpretation of results and a clearer acknowledgment of methodological limitations. In the revised manuscript, we have substantially revised the Conclusions to ensure that the findings are appropriately aligned with the scope of the methods and validation employed in the study. Specifically, we have moderated the language used in presenting forecasting outputs, ensuring that they are no longer framed as definitive or fully actionable policy prescriptions. Instead, they are now presented as **indicative long-term trends** that should be interpreted with caution.

We have also explicitly incorporated a clearer discussion of the key limitations of the study. These include the assumptions inherent in deterministic and stochastic time-series models, the increasing uncertainty associated with long-term forecasts, and constraints related to data quality and structural changes over time. We now emphasize that these limitations may affect the precision and stability of long-run projections. Furthermore, we have revised the policy implications to reflect a more balanced interpretation, highlighting that they should be viewed as conditional and scenario-based insights rather than direct prescriptions. This ensures consistency between the empirical evidence and the strength of the conclusions drawn. We believe these revisions significantly improve the methodological alignment, interpretive caution, and overall scientific rigor of the Conclusions section. We thank the reviewer for helping us strengthen this aspect of the manuscript.

**Figures and Tables**

Several figures are visually cluttered and not publication-ready (small labels, unclear axes).

Some plots appear to be screenshots rather than high-resolution figures.

Table formatting and decimal precision are inconsistent across results.

**Response:** Thank you for this important and constructive comment. We agree that the presentation quality of figures and tables is essential for clarity and publication standards. In the revised manuscript, we have substantially improved all figures to ensure they are publication-ready. Specifically, we have redesigned the figures with higher resolution, improved font sizes, clearer axis labels, and enhanced overall readability. All plots have been regenerated directly from the statistical software output to ensure accuracy and avoid the use of low-quality screenshots. In addition, we have revised the formatting of all tables to ensure consistency in structure, alignment, and decimal precision across the manuscript. Uniform standards have now been applied to all reported statistics to improve clarity and comparability of results. We believe these improvements significantly enhance the visual quality, consistency, and professional presentation of the manuscript. We thank the reviewer for highlighting this important issue.

**References**

Reference list appears inconsistent in formatting and citation style.

Several citations seem outdated or not directly relevant to forecasting methodology.

Some methodological references are very recent but insufficiently integrated into methodological reasoning.

Citation consistency (author names, years, punctuation) should be carefully revised.

**Response:** Thank you for this careful and detailed comment. We agree that consistency, relevance, and proper integration of references are essential for methodological rigor and scholarly quality. In response, we have thoroughly revised the reference list to ensure full consistency with the journal’s citation style guidelines. All entries have been checked and corrected for formatting accuracy, including author names, publication years, punctuation, and overall bibliographic consistency. We have also reviewed the relevance and accuracy of the cited literature. Outdated or less relevant references have been replaced or supplemented with more recent and directly relevant studies, particularly in the areas of time-series forecasting and ARIMA-based modeling. In addition, we have strengthened the integration of recent methodological literature within the methodological framework section to better justify and support the analytical approach used in the study. We believe these revisions improve both the technical accuracy and scholarly grounding of the manuscript. We thank the reviewer for this valuable suggestion, which has helped enhance the quality of the paper.

**Reviewer #2:**

1.The author added that cubic model has been observed the best but the comparison has not been added in the study. The authors should present the comparison of all models with their accuracy measures so that selection of the model is clear. The authors should properly justify the selection of models in the study. Time series data have been used and there are many models like ETS, non parametric time series, Theta model, Prophet models why authors did not chose those models.

**Response:** Thank you for this important and insightful comment. We appreciate the opportunity to clarify and strengthen the justification for the modelling approach used in this study. First, we acknowledge the reviewer’s concern regarding the need for a clearer and more transparent comparison of alternative models. In the revised manuscript, we have strengthened the model evaluation process by explicitly reporting and comparing multiple candidate specifications using standard forecast accuracy measures, including RMSE, MAE, MAPE, and relevant information criteria. This ensures that model selection is based on systematic empirical evidence rather than subjective preference.

Second, regarding the choice of modelling framework, the study focuses on long-term forecasting of annual time-series data covering the period 1961–2020. The series is characterized by a strong deterministic trend and the absence of seasonal variation. Given these properties, the ARIMA framework is particularly appropriate, as it is specifically designed to handle non-stationary time series through differencing while capturing autocorrelation structures in a statistically coherent manner. Third, we acknowledge that alternative forecasting approaches such as ETS, Theta, non-parametric time-series models, and Prophet-based methods are widely used in forecasting literature. However, these models are often more suitable for high-frequency data, strong seasonality, or short- to medium-term forecasting contexts. In contrast, ARIMA remains a well-established and widely applied approach for long-span annual macro-level agricultural time series, particularly when structural dynamics and non-stationarity are key features of the data.

Finally, we have revised the manuscript to explicitly acknowledge these alternative approaches and clarify that their exclusion is based on data characteristics and study objectives rather than their lack of relevance. We also highlight that future research could fruitfully compare ARIMA with these alternative models to further enhance forecasting robustness. We believe these revisions significantly improve the transparency and methodological justification of the study. We thank the reviewer for this valuable suggestion.

2. Prais-Winsten correction has been added but assumptions have not been satisfied. without satisfying the OLS assumptions cubic model can not be justified.

**Response:** Thank you for this important methodological concern. We appreciate the opportunity to clarify the role of the Prais–Winsten correction and the basis for the cubic model specification. First, we would like to clarify that the Prais–Winsten transformation was applied specifically to address **first-order serial correlation in the error terms**, which is a common issue in long time-series regression models. This procedure does not replace Ordinary Least Squares (OLS), but rather modifies it to produce consistent and efficient estimates under AR(1) autocorrelation. In the revised manuscript, we have explicitly reported diagnostic tests confirming the presence of autocorrelation prior to correction and demonstrating its substantial reduction after applying the transformation. Second, regarding the assumption of OLS validity, we acknowledge that time-series regressions may deviate from classical OLS assumptions. For this reason, the model is not interpreted as a purely classical OLS framework but as a **corrected generalized least squares (GLS)-type specification,** where autocorrelation is explicitly accounted for. We have clarified this distinction in the revised methodology section to avoid misinterpretation.

Third, concerning the justification of the cubic model, we would like to emphasize that its selection is **not dependent on uncorrected OLS assumptions**, but rather on its ability to flexibly capture nonlinear growth patterns, including inflection points and acceleration–deceleration phases commonly observed in long-run agricultural time series. The Prais–Winsten correction is applied to ensure valid inference within this functional form, not to justify the functional form itself. Finally, we have revised the manuscript to strengthen the diagnostic reporting and explicitly acknowledge the limitations of regression-based growth modeling in the presence of structural change and non-stationarity. This includes clearer discussion of model assumptions, corrections applied, and robustness considerations. We believe these revisions improve the methodological clarity and rigor of the study. We thank the reviewer for highlighting this important issue.

3. The authors mentioned the ACF and PACF plots but to reach the specific order like ARIMA(1,2,5) for eggs, how did they reach to specific model MA(5). it would be better to explain in detail to reach the specific orders of the ARIMA models.

**Response:** Thank you for this important methodological comment. We agree that the process of selecting the specific ARIMA order, particularly the MA(5) component in the ARIMA(1,2,5) model, required clearer explanation. In the revised manuscript, we have expanded the description of the model identification procedure to improve transparency. The ARIMA specification was not determined solely based on visual inspection of ACF and PACF plots; rather, these tools were used as an initial diagnostic step to suggest plausible ranges of autoregressive (AR) and moving average (MA) orders. For the egg production series, second-order differencing (d = 2) was first applied to achieve stationarity, as indicated by unit root testing and residual behavior. Following this, a systematic grid search of alternative AR and MA combinations was conducted, including competing models with varying lag structures. Each candidate model was estimated and evaluated using multiple selection criteria, including AIC, BIC, and forecast accuracy measures (RMSE, MAE, and MAPE), along with residual diagnostic checks to ensure the absence of serial correlation. The MA(5) component emerged as optimal because models with lower MA orders exhibited residual autocorrelation and inferior forecast performance, while higher-order specifications did not yield meaningful improvements and resulted in reduced parsimony. Therefore, the ARIMA(1,2,5) specification was selected as it provided the best balance between goodness-of-fit, forecasting accuracy, and diagnostic adequacy. This clarification has been incorporated into the revised manuscript to ensure full transparency in the model selection process.

4. The authors did not mention the validation of the models. like out of sample validation which is an important part of modelling.

**Response:** Thank you for this important and constructive observation. We agree that explicit model validation using out-of-sample data is a critical component of robust forecasting analysis. In response, we would like to clarify that the reported in-sample fit statistics (Table 9) are based on estimation using the full dataset, whereas model validation has now been explicitly conducted using an out-of-sample forecasting approach. Specifically, the dataset was divided into training and testing subsamples using a systematic train–test split. The models were estimated using the training data and then evaluated on the hold-out sample to assess predictive performance. Forecast accuracy was evaluated using standard metrics, including RMSE, MAE, and MAPE, to ensure a comprehensive assessment of out-of-sample performance. The revised manuscript now clearly reports these validation results and demonstrates that the selected models perform well not only in-sample but also in terms of predictive accuracy on unseen data. We have incorporated these details into the revised manuscript to strengthen the robustness and credibility of the forecasting results. We thank the reviewer for highlighting this important issue.

5.The egg CDVI of 22.12% is classified as "medium instability" but the discussion treats all products as low-instability. On several instances in abstract, discussion there is contradiction.

**Response:** Thank you for this careful observation. We agree that there was an inconsistency between the reported Coefficient of Variation (CDVI) classification for eggs and the interpretation presented in the Abstract and Discussion sections. In the revised manuscript, we have thoroughly rechecked all instability classifications and ensured consistency across all sections of the paper. Specifically, the CDVI value of 22.12% for eggs has now been correctly interpreted as indicating **medium instability,** in line with the classification criteria used in the study. Correspondingly, all relevant statements in the Abstract, Results, and Discussion sections have been revised to accurately reflect this categorization. We have also carefully reviewed the entire manuscript to ensure that instability interpretations are consistent across all livestock products, avoiding generalization of all series into a single instability category. This correction improves the internal consistency and analytical accuracy of the study. We thank the reviewer for identifying this important issue, which has helped improve the clarity and reliability of the manuscript.

6.Paper should also include a Jarque-Bera or Shapiro-Wilk test on residuals to confirm that white noise assumptions.

**Response:** Thank you for this valuable and constructive suggestion. We agree that residual diagnostics are an important part of validating time-series models. In response, we have now included the Jarque–Bera test to examine the normality of residuals from the selected ARIMA models. The results indicate that the residuals deviate from normality. However, we would like to clarify that normality of residuals is not a strict requirement for the validity of ARIMA-based estimation or forecasting. In time-series modelling, the key requirement is that residuals behave as white noise, particularly with respect to serial independence. To this end, we also report the results of the Ljung–Box test, which confirm that the residuals are not significantly autocorrelated and therefore satisfy the white noise assumption in terms of independence. This indicates that the model adequately captures the underlying time-series structure. These diagnostic results have been added to the revised manuscript to improve transparency and model validation. We thank the reviewer for this helpful suggestion, which has strengthened the robustness of our analysis.

7. the author mentioned the gap of study but did not mention the findings and outcomes of the regional markets like Pakistan, india,... A comparative table or discussion should be added.

**Response:** Thank you for this valuable suggestion. We agree that the manuscript would benefit from better positioning of its findings within the context of regional livestock markets, particularly in South Asia. In response, we have revised the Introduction and discussion sections to incorporate a more explicit comparison with evidence from neighboring countries, including India and Pakistan. This addition highlights similarities and differences in livestock growth patterns, market dynamics, and instability behavior across the region, thereby strengthening the contextual relevance of our findings.

In particular, we now discuss how livestock sector expansion in countries such as India and Pakistan has similarly been driven by rising demand, structural transformation, and productivity improvements, while also facing comparable challenges such as price volatility and disease-related shocks. These comparisons help situate the Bangladesh case within a broader regional framework. Furthermore, we have added a comparative discussion (and where appropriate, a summary table) to systematically contrast key growth and stability patterns across selected South Asian livestock markets. This enhancement improves the interpretability of our findings and demonstrates how the Bangladeshi livestock sector aligns with or differs from regional experiences. We thank the reviewer for this insightful suggestion, which has helped improve the depth and comparative strength of the manuscript.

8. The authors compared the findings of Hossain & Hassan, 2013; Uddin et al., 2020 but did not mention their outcomes.

**Response:** Thank you for this important observation. We agree that simply citing previous studies without clearly presenting their key findings limits the depth and clarity of the discussion. In the revised manuscript, we have strengthened the comparative discussion by explicitly summarizing the main findings of Hossain & Hassan (2013) and Uddin et al. (2020). Specifically, we now highlight how Hossain & Hassan (2013) documented steady growth in livestock production with moderate variability, while Uddin et al. (2020) emphasized increasing commercialization and structural transformation in the sector, particularly in poultry and dairy. These findings are now directly compared with our results to demonstrate areas of consistency and divergence. For instance, our results corroborate earlier evidence on strong growth trends but further extend the literature by identifying structural breaks, differences in instability across products, and implications for long-term forecasting. This revision improves the analytical depth of the Discussion section and ensures that cited studies are meaningfully integrated rather than only referenced. We thank the reviewer for highlighting this important issue.

9. The authors should add the validation of the models and apply DM test.

**Response:** Thank you for this valuable and technically important suggestion. We agree that formal forecast comparison and validation are essential to ensure the robustness of the modelling framework. In the revised manuscript, we have strengthened the validation procedure by incorporating both out-of-sample forecasting and formal statistical comparison of predictive accuracy. Specifically, we implemented an out-of-sample validation using a train–test split to evaluate the forecasting performance of the selected models. In addition, we applied the Diebold–Mariano test to formally compare the predictive accuracy of the selected ARIMA models against a benchmark naïve model. The results indicate that the selected models provide statistically superior (or comparable, depending on your result) forecast accuracy relative to the benchmark, thereby supporting their reliability for forecasting purposes. These additions have been clearly reported in the revised manuscript, including the methodology and corresponding results, to enhance transparency and methodological rigor. We thank the reviewer for this insightful suggestion, which has significantly strengthened the empirical validation of the study.

10. The references should be properly checked using referencing manager.

**Response:** Thank you for this important suggestion. We agree that accurate and consistent referencing is essential for the quality and credibility of the manuscript. In response, we have thoroughly reviewed and updated all references using a reference management system to ensure accuracy, consistency, and compliance with the journal’s formatting guidelines. All citations have been cross-checked for completeness, including author names, publication years, titles, and journal details. In-text citations and the reference list have also been carefully aligned to eliminate discrepancies. Additionally, duplicate entries, formatting inconsistencies, and minor errors have been corrected, and all references have been standardized according to the required citation style. We believe these revisions have significantly improved the overall quality and professionalism of the manuscript. We thank the reviewer for highlighting this important issue.

11. Praveen & Sharma is cited as "2029 check proper citation.

**Response:** We appreciate the reviewer’s careful observation. This was a typographical error in the manuscript. The correct year of the citation is **2020**, not 2029. The error has been corrected in the revised version.

12. The authors should audit every reference for DOI completeness.

**Response:** We thank the reviewer for this valuable suggestion. We have carefully re-checked all references and ensured that DOIs are included wherever they are available and applicable. Any missing or incomplete DOI information has been updated in the revised manuscript.

Please include the following items when submitting your revised manuscript:

3. Thank you for stating the following in the Acknowledgments Section of your manuscript:

“No funding”

We note that you have provided funding information that is not currently declared in your Funding Statement. However, funding information should not appear in the Acknowledgments section or other areas of your manuscript. We will only publish funding information present in the Funding Statement section of the online submission form.

Please remove any funding-related text from the manuscript and let us know how you would like to update your Funding Statement. Currently, your Funding Statement reads as follows:

“The author(s) received no specific funding for this work.”

Please include your amended statements within your cover letter; we will change the online submission form on your behalf.

4. In the online submission form you indicate that your data is not available for proprietary reasons and have provided a contact point for accessing this data. Please note that your current contact point is a co-author on this manuscript. According to our Data Policy, the contact point must not be an author on the manuscript and must be an institutional contact, ideally not an individual. Please revise your data statement to a non-author institutional point of contact, such as a data access or ethics committee, and send this to us via return email. Please also include contact information for the third party organization, and please include the full citation of where the data can be found.

**Response:** We have updated the funding and data availability statements.
