# Supplementary material for: Growth, instability and future outlook of livestock population and products in Bangladesh
Source: PLoS One. 2026 Jun 26;21(6):e0351876. doi: 10.1371/journal.pone.0351876 (PMC13308833; doi:10.1371/journal.pone.0351876)
Supplement: Attachment — Submitted filename: Response_to_Reviewers_auresp_2.docx [file pone.0351876.s005.docx]

PONE-D-26-07519R1

**Growth, Instability and Future Outlook of Livestock Population and Products in Bangladesh**

**Editor Comments**

1. The authors completely failed to update the abstract to match their revised forecasting data. In the abstract, they claim that if the current trend continues, egg production will reach 4,646,447 MT in 2041 (a 582% increase). In Table 10 (Forecasting result) and Table 11 (Projected demand and production), the 2041 ARIMA forecast for eggs is actually reported as 1,289,863 MT. The track-changes section reveals the authors updated their models and changed the table data from ~4.6 million to ~1.28 million, but they forgot to update the core conclusions in the abstract. This means the paper's primary executive summary contains wildly inaccurate data based on their own revised calculations.

**Response:** We sincerely thank the Editor for identifying this important inconsistency and apologize for the oversight. During the previous revision stage, the forecasting analysis was re-estimated using R programming instead of SPSS, which resulted in updated forecast values, particularly for egg production. Although the forecast values in the relevant tables were revised accordingly, the corresponding figures reported in the abstract were inadvertently not updated. Following the Editor’s comment, we carefully reviewed the entire manuscript and reran all forecasting models to verify the results and ensure consistency throughout. The abstract has now been corrected to reflect the revised ARIMA forecast. In addition, all related tables, figures, projected demand estimates, and discussion sections have been thoroughly checked and updated where necessary to ensure that the reported values and interpretations are fully consistent across the manuscript.

2. Reviewer 1 explicitly stated: "Forecast values are presented without confidence intervals or prediction uncertainty, which is critical for forecasting studies." In their cover letter and rebuttal, the authors claim, "We have added out-of-sample validation, forecast intervals, and Diebold–Mariano tests..." There are no forecast intervals provided anywhere in the text, tables, or figures. Table 10 provides only singular point estimates for the 21-year forecast. Presenting a 21-year economic forecast without 95% confidence intervals/prediction bounds is a severe methodological oversight, especially since they explicitly told you they fixed it.

**Response:** We appreciate the Editor’s careful observation regarding the omission of prediction intervals in the revised manuscript. We agree that reporting forecast uncertainty is essential, particularly for long-term projections, and we appreciate the Editor’s comment. The Editor is correct that although forecast intervals were generated during the analysis stage, they were inadvertently not presented in the manuscript tables and figures.

To address this issue, we have now explicitly incorporated 95% prediction intervals for all ARIMA forecasts (2021–2041). The revised manuscript now includes forecast uncertainty measures in both tabular and graphical formats. Specifically, Appendix Table A6 presents the forecasted values together with lower and upper 95% prediction bounds for milk, meat, and egg production, while the revised forecasting figures now display prediction interval bands around the forecast trajectories. Additionally, because the forecasting models were re-estimated using R programming, slight revisions occurred in the projected values for milk and meat production, and these updated estimates have been consistently incorporated throughout the manuscript.

3. In the Methodology section, the authors justify splitting their 60-year data into three sub-periods by stating they conducted an ANOVA test that "confirmed statistically significant differences." However, they do not report the actual F-statistics, degrees of freedom, or exact p-values anywhere in the manuscript to prove this.

**Response:** Thank you for highlighting this important issue. In the revised manuscript, we have now explicitly reported the ANOVA results used to justify the division of the 60-year time series into three sub-periods. The Methodology section has been updated to include the relevant F-statistics, degrees of freedom, and exact p-values for the key livestock production variables. To provide full transparency, the complete ANOVA results are also presented in **Appendix Table A7**, which reports the test statistics for milk, meat, and egg production across the three study periods. These results confirm that the mean production levels differ significantly across the sub-periods, thereby supporting the temporal segmentation adopted in the analysis.

4. Reviewer 2 asked for a normality test on the residuals. The authors added it (Appendix Table A3) and found the residuals are highly non-normal (p=0.000). They dismiss this by saying ARIMA only requires white noise (no autocorrelation). While true for generating point estimates, non-normal residuals heavily impact the calculation of confidence intervals which makes their failure to provide those confidence intervals (Point #2 above) even more problematic.

**Response:** We thank the Editor for this valuable observation. We acknowledge that the non-normality of residuals may influence the precision of interval estimation in long-term forecasting models. To address this concern, we have now explicitly incorporated 95% prediction intervals into the revised manuscript. We additionally note that although residual normality was not fully satisfied, the ARIMA models exhibited residual white-noise properties based on Ljung–Box diagnostics, indicating the absence of significant autocorrelation and supporting the adequacy of the forecasting models for trend estimation.

5. While your revised manuscript provides a strong macroeconomic forecast of livestock and poultry production, your discussion regarding structural constraints, specifically disease vulnerability and food safety, lacks sufficient regional context from South Asia. To strengthen your discussion on the specific bottlenecks limiting the livestock and dairy sectors, please incorporate the following relevant references into your manuscript during this revision:

I. Khan, R. S. A., Sajid, S., Habib, M., Ali, W., Shah, M. S. D., & Sarfraz, M. (2017). History of Gumboro (infectious bursal disease) in Pakistan. Saudi Pharmaceutical Journal, 25(4), 453-459.
Note to authors: Please integrate this citation into the Discussion section where you address the "medium instability" of egg production, utilizing it to highlight how viral disease outbreaks severely impact poultry sector stability in the region.

II. Sanaullah, S., Sajjad, R., Sehrish, N., & IrfanUllah, K. (2022). Emergence, existence and distribution of foot and mouth disease in Pakistan in comparison with the global perspective. GSC Biological and Pharmaceutical Sciences, 7(1), 102-110.
Note to authors: Please add this reference when discussing the structural bottlenecks and poor veterinary coverage limiting cattle and meat production to provide a concrete example of major regional disease constraints.

III. Gohar, S., Abbas, G., & Sanaullah, S. (2017). Prevalence and antimicrobial resistance of Listeria monocytogenes isolated from raw milk and dairy products. Zibeline International Publishing, 1(1), 10-14.
Note to authors: This should be cited when discussing the rapid commercialization of the dairy/milk sector and the resulting necessity for improved processing, cold-chain infrastructure, and pathogen/food safety control.

Please ensure these are naturally integrated into the flow of your discussion section. I look forward to seeing these updates in your next revision.

**Response:** We sincerely thank the Editor for these valuable suggestions and relevant references. Following the recommendation, we carefully incorporated the suggested studies into the discussion section to strengthen the regional context of livestock sector constraints in South Asia. These references have been integrated into the narrative to provide additional empirical support and regional context for the structural bottlenecks identified in our analysis. We believe that these additions substantially strengthen the discussion and enhance the policy relevance of the manuscript. We are grateful to the Editor for these valuable recommendations.
