# Supplementary material for: Growth, instability and future outlook of livestock population and products in Bangladesh
Source: PLoS One. 2026 Jun 26;21(6):e0351876. doi: 10.1371/journal.pone.0351876 (PMC13308833; doi:10.1371/journal.pone.0351876)
Supplement: Peer review history

# Peer review: Decision Letter 0

**DOI:** 10.1371/journal.pone.0351876.r001

**Authors:** Sanaullah Sajid

27 Mar 2026

PONE-D-26-07519

Growth, Instability and Forecasting of Livestock Population and Products in Bangladesh: Analysis Using Best Fitting Deterministic and Stochastic Models

PLOS One

Dear Dr. Islam,

Thank you for submitting your manuscript to PLOS ONE. After careful consideration, we feel that it has merit but does not fully meet PLOS ONE’s publication criteria as it currently stands. Therefore, we invite you to submit a revised version of the manuscript that addresses the points raised during the review process.

Please submit your revised manuscript by May 11 2026 11:59PM. If you will need more time than this to complete your revisions, please reply to this message or contact the journal office at plosone@plos.org. When you're ready to submit your revision, log on to https://www.editorialmanager.com/pone/ and select the 'Submissions Needing Revision' folder to locate your manuscript file.

Please include the following items when submitting your revised manuscript:

- A letter that responds to each point raised by the academic editor and reviewer(s). You should upload this letter as a separate file labeled 'Response to Reviewers'.
- A marked-up copy of your manuscript that highlights changes made to the original version. You should upload this as a separate file labeled 'Revised Manuscript with Track Changes'.
- An unmarked version of your revised paper without tracked changes. You should upload this as a separate file labeled 'Manuscript'.

If you would like to make changes to your financial disclosure, please include your updated statement in your cover letter. Guidelines for resubmitting your figure files are available below the reviewer comments at the end of this letter.

If applicable, we recommend that you deposit your laboratory protocols in protocols.io to enhance the reproducibility of your results. Protocols.io assigns your protocol its own identifier (DOI) so that it can be cited independently in the future. For instructions see: https://journals.plos.org/plosone/s/submission-guidelines#loc-laboratory-protocols. Additionally, PLOS ONE offers an option for publishing peer-reviewed Lab Protocol articles, which describe protocols hosted on protocols.io. Read more information on sharing protocols at https://plos.org/protocols?utm_medium=editorial-email&utm_source=authorletters&utm_campaign=protocols.

As the corresponding author, your ORCID iD is verified in the submission system and will appear in the published article. PLOS supports the use of ORCID, and we encourage all coauthors to register for an ORCID iD and use it as well. Please encourage your coauthors to verify their ORCID iD within the submission system before final acceptance, as unverified ORCID iDs will not appear in the published article. *Only*  the individual author can complete the verification step; PLOS staff *cannot*  verify ORCID iDs on behalf of authors.

We look forward to receiving your revised manuscript.

Kind regards,

Sanaullah Sajid, Ph.D.

Academic Editor

PLOS One

Journal Requirements:

When submitting your revision, we need you to address these additional requirements.

1. Please ensure that your manuscript meets PLOS ONE's style requirements, including those for file naming. The PLOS ONE style templates can be found at

https://journals.plos.org/plosone/s/file?id=wjVg/PLOSOne_formatting_sample_main_body.pdf and

https://journals.plos.org/plosone/s/file?id=ba62/PLOSOne_formatting_sample_title_authors_affiliations.pdf

2. Please update your submission to use the PLOS LaTeX template. The template and more information on our requirements for LaTeX submissions can be found at http://journals.plos.org/plosone/s/latex.

3. Thank you for stating the following in the Acknowledgments Section of your manuscript:

“No funding”

We note that you have provided funding information that is not currently declared in your Funding Statement. However, funding information should not appear in the Acknowledgments section or other areas of your manuscript. We will only publish funding information present in the Funding Statement section of the online submission form.

Please remove any funding-related text from the manuscript and let us know how you would like to update your Funding Statement. Currently, your Funding Statement reads as follows:

“The author(s) received no specific funding for this work.”

Please include your amended statements within your cover letter; we will change the online submission form on your behalf.

4. In the online submission form you indicate that your data is not available for proprietary reasons and have provided a contact point for accessing this data. Please note that your current contact point is a co-author on this manuscript. According to our Data Policy, the contact point must not be an author on the manuscript and must be an institutional contact, ideally not an individual. Please revise your data statement to a non-author institutional point of contact, such as a data access or ethics committee, and send this to us via return email. Please also include contact information for the third party organization, and please include the full citation of where the data can be found.

5. If the reviewer comments include a recommendation to cite specific previously published works, please review and evaluate these publications to determine whether they are relevant and should be cited. There is no requirement to cite these works unless the editor has indicated otherwise.

[Note: HTML markup is below. Please do not edit.]

Reviewers' comments:

Reviewer's Responses to Questions

**Comments to the Author**

1. Is the manuscript technically sound, and do the data support the conclusions?

The manuscript must describe a technically sound piece of scientific research with data that supports the conclusions. Experiments must have been conducted rigorously, with appropriate controls, replication, and sample sizes. The conclusions must be drawn appropriately based on the data presented.

Reviewer #1: Partly

Reviewer #2: Partly

**********

2. Has the statistical analysis been performed appropriately and rigorously?

Reviewer #1: No

Reviewer #2: No

**********

3. Have the authors made all data underlying the findings in their manuscript fully available?

The PLOS Data policy requires authors to make all data underlying the findings described in their manuscript fully available without restriction, with rare exception (please refer to the Data Availability Statement in the manuscript PDF file). The data should be provided as part of the manuscript or its supporting information, or deposited to a public repository. For example, in addition to summary statistics, the data points behind means, medians and variance measures should be available. If there are restrictions on publicly sharing data—e.g. participant privacy or use of data from a third party—those must be specified.

Reviewer #1: No

Reviewer #2: Yes

**********

4. Is the manuscript presented in an intelligible fashion and written in standard English?

PLOS ONE does not copyedit accepted manuscripts, so the language in submitted articles must be clear, correct, and unambiguous. Any typographical or grammatical errors should be corrected at revision, so please note any specific errors here.

Reviewer #1: No

Reviewer #2: Yes

**********

5. Review Comments to the Author

Please use the space provided to explain your answers to the questions above. You may also include additional comments for the author, including concerns about dual publication, research ethics, or publication ethics. (Please upload your review as an attachment if it exceeds 20,000 characters)

Reviewer #1: Title

The title is excessively long and contains methodological detail (“Best Fitting Deterministic and Stochastic Models”) that reads more like methods than a research focus.

The phrase “Best Fitting” is interpretative and should be avoided unless rigorously justified against alternative frameworks.

The title could be simplified to improve clarity and readability without loss of scientific meaning.

Abstract

The abstract reports many numerical results but lacks clear emphasis on methodological limitations and assumptions underlying long-term forecasting (to 2041).

The statement that outputs are “an invaluable resource” is overstated and not supported by validation or uncertainty assessment.

Forecast values are presented without confidence intervals or prediction uncertainty, which is critical for forecasting studies.

The abstract implies robustness but does not mention model validation strategy.

Introduction

The introduction is overly descriptive and contains extensive background statistics that do not directly build the research gap.

The literature gap is weakly justified; claims of scarcity of studies are not sufficiently supported by systematic referencing.

Some statements are outdated or inconsistently referenced, and several citations appear disconnected from the forecasting objective.

The rationale for combining deterministic growth models with ARIMA is stated but not theoretically justified.

Methodology

Data

Data source is limited to FAOSTAT without discussion of potential data quality issues or structural breaks over a 60-year period.

No explanation is provided for handling missing values, revisions, or consistency across historical data series.

Splitting the time series into three sub-periods appears arbitrary; no statistical justification is provided.

Growth Models

Model selection is based heavily on goodness-of-fit statistics (R², RMSE, etc.), which may favor overfitting (especially cubic models) in long-term trend data.

The theoretical justification for preferring cubic growth models over alternatives is insufficient.

Growth rate derivation from the cubic function is presented mathematically but lacks interpretation of economic or biological meaning.

Autocorrelation Treatment

The use of Prais–Winsten transformation for growth models is reported, but the rationale for applying it alongside ARIMA (which already accounts for autocorrelation) is unclear.

Durbin–Watson statistics reported before transformation are extremely low, suggesting severe autocorrelation that may indicate model misspecification rather than simply a correction issue.

Forecasting / ARIMA

Model identification relies largely on visual ACF/PACF interpretation; a clearer systematic model selection procedure is needed.

Forecast validation (e.g., train–test split, out-of-sample testing) is not described.

Forecast horizon to 2041 (21 years) is long relative to model structure and lacks uncertainty discussion.

Egg series stationarity remains borderline after differencing (p = 0.08), yet ARIMA modeling proceeds without sufficient justification.

Results

Descriptive Statistics

Some descriptive statistics appear inconsistent (e.g., mean/SD scales and formatting), suggesting possible data reporting issues.

Interpretation of skewness and kurtosis is superficial and does not contribute meaningfully to later modeling.

Growth and Instability Analysis

Results are largely descriptive without statistical testing of differences between periods.

The interpretation of instability categories (low/medium/high) lacks discussion of practical implications.

Figures are difficult to interpret due to scaling and presentation style.

Forecasting Results

Model comparison relies heavily on in-sample fit metrics; no predictive performance evaluation is shown.

Residual diagnostics are described qualitatively but lack formal statistical tests (e.g., Ljung–Box).

Forecast plots and uncertainty bands are missing, limiting interpretability of future projections.

Discussion

Discussion mainly restates results rather than critically interpreting findings.

Limited comparison with existing forecasting studies beyond descriptive references.

No discussion of structural changes (policy shifts, disease outbreaks, climate factors) that could invalidate long-term forecasts.

Policy implications are presented as definitive despite methodological uncertainty.

Conclusions

Conclusions are stronger than warranted by the methods and validation presented.

Forecast outputs are framed as actionable policy guidance without acknowledging model limitations.

Key limitations (model assumptions, uncertainty, data constraints) are insufficiently emphasized.

Figures and Tables

Several figures are visually cluttered and not publication-ready (small labels, unclear axes).

Some plots appear to be screenshots rather than high-resolution figures.

Table formatting and decimal precision are inconsistent across results.

References

Reference list appears inconsistent in formatting and citation style.

Several citations seem outdated or not directly relevant to forecasting methodology.

Some methodological references are very recent but insufficiently integrated into methodological reasoning.

Citation consistency (author names, years, punctuation) should be carefully revised.

Reviewer #2: i have the following recommendations for the study.

1.The author added that cubic model has been observed the best but the comparison has not been added in the study. The authors should present the comparison of all models with their accuracy measures so that selection of the model is clear. The authors should properly justify the selection of models in the study. Time series data have been used and there are many models like ETS, non parametric time series, Theta model, Prophet models why authors did not chose those models.

2. Prais-Winsten correction has been added but assumptions have not been satisfied. without satisfying the OLS assumptions cubic model can not be justified.

3. The authors mentioned the ACF and PACF plots but to reach the specific order like ARIMA(1,2,5) for eggs, how did they reach to specific model MA(5). it would be better to explain in detail to reach the specific orders of the ARIMA models.

4. The authors did not mention the validation of the models. like out of sample validation which is an important part of modelling.

5.The egg CDVI of 22.12% is classified as "medium instability" but the discussion treats all products as low-instability. On several instances in abstract, discussion there is contradiction.

6.Paper should also include a Jarque-Bera or Shapiro-Wilk test on residuals to confirm that white noise assumptions.

7. the author mentioned the gap of study but did not mention the findings and outcomes of the regional markets like Pakistan, india,... A comparative table or discussion should be added.

8. The authors compared the findings of Hossain & Hassan, 2013; Uddin et al., 2020 but did not mention their outcomes.

9. The authors should add the validation of the models,and apply DM test.

10. The references should be properly checked using referencing manager.

11. Praveen & Sharma is cited as "2029 check proper citation.

12. The authors should audit every reference for DOI completeness.

**********

6. PLOS authors have the option to publish the peer review history of their article (what does this mean?). If published, this will include your full peer review and any attached files.

If you choose “no”, your identity will remain anonymous but your review may still be made public.

**Do you want your identity to be public for this peer review?**  For information about this choice, including consent withdrawal, please see our Privacy Policy.

Reviewer #1: No

Reviewer #2: No

**********

[NOTE: If reviewer comments were submitted as an attachment file, they will be attached to this email and accessible via the submission site. Please log into your account, locate the manuscript record, and check for the action link "View Attachments". If this link does not appear, there are no attachment files.]

To ensure your figures meet our technical requirements, please review our figure guidelines: https://journals.plos.org/plosone/s/figures

You may also use PLOS’s free figure tool, NAAS, to help you prepare publication quality figures: https://journals.plos.org/plosone/s/figures#loc-tools-for-figure-preparation.

NAAS will assess whether your figures meet our technical requirements by comparing each figure against our figure specifications.

Attachment Submitted filename: reviewer comments.docx

# Author comment: Author response to Decision Letter 1

**DOI:** 10.1371/journal.pone.0351876.r002

30 Apr 2026

Attached herewith

Attachment Submitted filename: Response to Reviewers.docx

# Editor report: Decision Letter 1

**DOI:** 10.1371/journal.pone.0351876.r003

**Authors:** Sanaullah Sajid, Sanaullah Sajid

13 May 2026

PONE-D-26-07519R1

Growth, Instability and Future Outlook of Livestock Population and Products in Bangladesh

PLOS One

Dear Dr. Islam,

Thank you for submitting your manuscript to PLOS ONE. After careful consideration, we feel that it has merit but does not fully meet PLOS ONE’s publication criteria as it currently stands. Therefore, we invite you to submit a revised version of the manuscript that addresses the points raised during the review process.

Please submit your revised manuscript by Jun 27 2026 11:59PM. If you will need more time than this to complete your revisions, please reply to this message or contact the journal office at plosone@plos.org. When you're ready to submit your revision, log on to https://www.editorialmanager.com/pone/ and select the 'Submissions Needing Revision' folder to locate your manuscript file.

Please include the following items when submitting your revised manuscript:

- A letter that responds to each point raised by the academic editor and reviewer(s). You should upload this letter as a separate file labeled 'Response to Reviewers'.
- A marked-up copy of your manuscript that highlights changes made to the original version. You should upload this as a separate file labeled 'Revised Manuscript with Track Changes'.
- An unmarked version of your revised paper without tracked changes. You should upload this as a separate file labeled 'Manuscript'.

If you would like to make changes to your financial disclosure, please include your updated statement in your cover letter. Guidelines for resubmitting your figure files are available below the reviewer comments at the end of this letter.

If applicable, we recommend that you deposit your laboratory protocols in protocols.io to enhance the reproducibility of your results. Protocols.io assigns your protocol its own identifier (DOI) so that it can be cited independently in the future. For instructions see: https://journals.plos.org/plosone/s/submission-guidelines#loc-laboratory-protocols. Additionally, PLOS ONE offers an option for publishing peer-reviewed Lab Protocol articles, which describe protocols hosted on protocols.io. Read more information on sharing protocols at https://plos.org/protocols?utm_medium=editorial-email&utm_source=authorletters&utm_campaign=protocols.

As the corresponding author, your ORCID iD is verified in the submission system and will appear in the published article. PLOS supports the use of ORCID, and we encourage all coauthors to register for an ORCID iD and use it as well. Please encourage your coauthors to verify their ORCID iD within the submission system before final acceptance, as unverified ORCID iDs will not appear in the published article. *Only*  the individual author can complete the verification step; PLOS staff *cannot*  verify ORCID iDs on behalf of authors.

We look forward to receiving your revised manuscript.

Kind regards,

Sanaullah Sajid, Ph.D.

Academic Editor

PLOS One

Journal Requirements:

If the reviewer comments include a recommendation to cite specific previously published works, please review and evaluate these publications to determine whether they are relevant and should be cited. There is no requirement to cite these works unless the editor has indicated otherwise.

Additional Editor Comments:

1. The authors completely failed to update the abstract to match their revised forecasting data. In the abstract, they claim that if the current trend continues, egg production will reach 4,646,447 MT in 2041 (a 582% increase). In Table 10 (Forecasting result) and Table 11 (Projected demand and production), the 2041 ARIMA forecast for eggs is actually reported as 1,289,863 MT. The track-changes section reveals the authors updated their models and changed the table data from ~4.6 million to ~1.28 million, but they forgot to update the core conclusions in the abstract. This means the paper's primary executive summary contains wildly inaccurate data based on their own revised calculations.

2. Reviewer 1 explicitly stated: "Forecast values are presented without confidence intervals or prediction uncertainty, which is critical for forecasting studies." In their cover letter and rebuttal, the authors claim, "We have added out-of-sample validation, forecast intervals, and Diebold–Mariano tests..." There are no forecast intervals provided anywhere in the text, tables, or figures. Table 10 provides only singular point estimates for the 21-year forecast. Presenting a 21-year economic forecast without 95% confidence intervals/prediction bounds is a severe methodological oversight, especially since they explicitly told you they fixed it.

3. In the Methodology section, the authors justify splitting their 60-year data into three sub-periods by stating they conducted an ANOVA test that "confirmed statistically significant differences." However, they do not report the actual F-statistics, degrees of freedom, or exact p-values anywhere in the manuscript to prove this.

4. Reviewer 2 asked for a normality test on the residuals. The authors added it (Appendix Table A3) and found the residuals are highly non-normal (p=0.000). They dismiss this by saying ARIMA only requires white noise (no autocorrelation). While true for generating point estimates, non-normal residuals heavily impact the calculation of confidence intervals which makes their failure to provide those confidence intervals (Point #2 above) even more problematic.

5. While your revised manuscript provides a strong macroeconomic forecast of livestock and poultry production, your discussion regarding structural constraints, specifically disease vulnerability and food safety, lacks sufficient regional context from South Asia. To strengthen your discussion on the specific bottlenecks limiting the livestock and dairy sectors, please incorporate the following relevant references into your manuscript during this revision:

I. Khan, R. S. A., Sajid, S., Habib, M., Ali, W., Shah, M. S. D., & Sarfraz, M. (2017). History of Gumboro (infectious bursal disease) in Pakistan. Saudi Pharmaceutical Journal, 25(4), 453-459.

Note to authors: Please integrate this citation into the Discussion section where you address the "medium instability" of egg production, utilizing it to highlight how viral disease outbreaks severely impact poultry sector stability in the region.

II. Sanaullah, S., Sajjad, R., Sehrish, N., & IrfanUllah, K. (2022). Emergence, existence and distribution of foot and mouth disease in Pakistan in comparison with the global perspective. GSC Biological and Pharmaceutical Sciences, 7(1), 102-110.

Note to authors: Please add this reference when discussing the structural bottlenecks and poor veterinary coverage limiting cattle and meat production to provide a concrete example of major regional disease constraints.

III. Gohar, S., Abbas, G., & Sanaullah, S. (2017). Prevalence and antimicrobial resistance of Listeria monocytogenes isolated from raw milk and dairy products. Zibeline International Publishing, 1(1), 10-14.

Note to authors: This should be cited when discussing the rapid commercialization of the dairy/milk sector and the resulting necessity for improved processing, cold-chain infrastructure, and pathogen/food safety control.

Please ensure these are naturally integrated into the flow of your discussion section. I look forward to seeing these updates in your next revision.

[Note: HTML markup is below. Please do not edit.]

[NOTE: If reviewer comments were submitted as an attachment file, they will be attached to this email and accessible via the submission site. Please log into your account, locate the manuscript record, and check for the action link "View Attachments". If this link does not appear, there are no attachment files.]

To ensure your figures meet our technical requirements, please review our figure guidelines: https://journals.plos.org/plosone/s/figures

You may also use PLOS’s free figure tool, NAAS, to help you prepare publication quality figures: https://journals.plos.org/plosone/s/figures#loc-tools-for-figure-preparation.

NAAS will assess whether your figures meet our technical requirements by comparing each figure against our figure specifications.

# Author comment: Author response to Decision Letter 2

**DOI:** 10.1371/journal.pone.0351876.r004

29 May 2026

Response to reviewer's letter included

Attachment Submitted filename: Response_to_Reviewers_auresp_2.docx

# Editor report: Decision Letter 2

**DOI:** 10.1371/journal.pone.0351876.r005

**Authors:** Sanaullah Sajid, Sanaullah Sajid, Sanaullah Sajid

2 Jun 2026

Growth, Instability and Future Outlook of Livestock Population and Products in Bangladesh

PONE-D-26-07519R2

Dear Dr. Islam,

We’re pleased to inform you that your manuscript has been judged scientifically suitable for publication and will be formally accepted for publication once it meets all outstanding technical requirements.

Within one week, you’ll receive an e-mail detailing the required amendments. When these have been addressed, you’ll receive a formal acceptance letter and your manuscript will be scheduled for publication.

An invoice will be generated when your article is formally accepted. Please note, if your institution has a publishing partnership with PLOS and your article meets the relevant criteria, all or part of your publication costs will be covered. Please make sure your user information is up-to-date by logging into Editorial Manager at Editorial Manager® and clicking the ‘Update My Information' link at the top of the page. For questions related to billing, please contact billing support.

If your institution or institutions have a press office, please notify them about your upcoming paper to help maximize its impact. If they’ll be preparing press materials, please inform our press team as soon as possible -- no later than 48 hours after receiving the formal acceptance. Your manuscript will remain under strict press embargo until 2 pm Eastern Time on the date of publication. For more information, please contact onepress@plos.org.

Kind regards,

Sanaullah Sajid, Ph.D.

Academic Editor

PLOS One

Additional Editor Comments (optional):

Reviewers' comments:

# Editor report: Acceptance letter

**DOI:** 10.1371/journal.pone.0351876.r006

**Authors:** Sanaullah Sajid, Sanaullah Sajid, Sanaullah Sajid

PONE-D-26-07519R2

PLOS One

Dear Dr. Islam,

I'm pleased to inform you that your manuscript has been deemed suitable for publication in PLOS One. Congratulations! Your manuscript is now being handed over to our production team.

At this stage, our production department will prepare your paper for publication. This includes ensuring the following:

* All references, tables, and figures are properly cited

* All relevant supporting information is included in the manuscript submission,

* There are no issues that prevent the paper from being properly typeset

You will receive further instructions from the production team, including instructions on how to review your proof when it is ready. Please keep in mind that we are working through a large volume of accepted articles, so please give us a few days to review your paper and let you know the next and final steps.

Lastly, if your institution or institutions have a press office, please let them know about your upcoming paper now to help maximize its impact. If they'll be preparing press materials, please inform our press team within the next 48 hours. Your manuscript will remain under strict press embargo until 2 pm Eastern Time on the date of publication. For more information, please contact onepress@plos.org.

You will receive an invoice from PLOS for your publication fee after your manuscript has reached the completed accept phase. If you receive an email requesting payment before acceptance or for any other service, this may be a phishing scheme. Learn how to identify phishing emails and protect your accounts at https://explore.plos.org/phishing.

If we can help with anything else, please email us at customercare@plos.org.

Thank you for submitting your work to PLOS ONE and supporting open access.

Kind regards,

PLOS ONE Editorial Office Staff

on behalf of

Dr. Sanaullah Sajid

Academic Editor

PLOS One
